# Designing a multi-epitope vaccine to provoke the robust immune response against influenza A H7N9

**DOI:** 10.1038/s41598-021-03932-2

**Published:** 2021-12-29

**Authors:** Hossein Tarrahimofrad, Somayyeh Rahimnahal, Javad Zamani, Ehsan Jahangirian, Saeed Aminzadeh

**Affiliations:** 1 Bioprocess Engineering Group, Institute of Industrial and Environmental Biotechnology, National Institute of Genetic Engineering and Biotechnology (NIGEB), Tehran, Iran; 2 Department of Animal Science, Faculty of Agriculture, Ilam University, Ilam, Iran

**Keywords:** Biochemistry, Biotechnology, Computational biology and bioinformatics, Immunology, Molecular biology

## Abstract

A new strain of Influenza A Virus (IAV), so-called "H7N9 Avian Influenza", is the first strain of this virus in which a human is infected by transmitting the N9 of influenza virus. Although continuous human-to-human transmission has not been reported, the occurrence of various H7N9-associated epidemics and the lack of production of strong antibodies against H7N9 in humans warn of the potential for H7N9 to become a new pandemic. Therefore, the need for effective vaccination against H7N9 as a life-threatening viral pathogen has become a major concern. The current study reports the design of a multi-epitope vaccine against Hemagglutinin (HA) and Neuraminidase (NA) proteins of H7N9 Influenza A virus by prediction of Cytotoxic T lymphocyte (CTL), Helper T lymphocyte (HTL), IFN-γ and B-cell epitopes. Human β-defensin-3 (HβD-3) and pan HLA DR-binding epitope (PADRE) sequence were considered as adjuvant. EAAAK, AAY, GPGPG, HEYGAEALERAG, KK and RVRR linkers were used as a connector for epitopes. The final construct contained 777 amino acids that are expected to be a recombinant protein of about ~ 86.38 kDa with antigenic and non-allergenic properties after expression. Modeled protein analysis based on the tertiary structure validation, docking studies, and molecular dynamics simulations results like Root-mean-square deviation (RMSD), Gyration, Root-mean-square fluctuation (RMSF) and Molecular Mechanics Poisson-Boltzmann Surface Area (MM/PBSA) showed that this protein has a stable construct and capable of being in interaction with Toll-like receptor 7 (TLR7), TLR8 and m826 antibody. Analysis of the obtained data the demonstrates that suggested vaccine has the potential to induce the immune response by stimulating T and Bcells, and may be utilizable for prevention purposes against Avian Influenza A (H7N9).

## Introduction

In February 2013, for the first time in China, a human infection associated with the new avian influenza A (H7N9) virus was reported. The new Asian lineage avian-origin influenza A (H7N9) virus has a higher infection risk for humans^1^. Since then, H7N9 infections have continued to occur during five waves of epidemics. The virus has caused increased mortality in any epidemic periodic by causing acute respiratory distress syndrome, especially in the elderly^2,3^.

Influenza A virus (IAV) is a negative single-strand RNA (ssRNA) virus that belongs to the family Orthomyxoviridae. Eight gene segments include HA, NA, Nuclear export protein (NEP), Matrix protein 2 (M2), Nucleocapsid protein (NP), Polymerase PA (PA), Polymerase PB1 (PB1) and Polymerase PB2 (PB2) in the genome, approximately 13,000 bp of IAVs, are responsible for encoding about 17 different types of proteins^4,5^. Studies have shown that the H7N9 influenza virus originated probably from the reassortment gene so that its surface genes such as HA and NA related to migratory birds and other internal six genes come from H9N2 avian influenza^6^. In general, gene reassortment, mutation, and recombination are considered as evolution mechanisms in the RNA viruses such as influenza which distinguishes between different strains of influenza^7–9^. HA glycoprotein antigen is known as one of the H7N9 major envelope proteins that are divided into two subunits of HA1 and HA2. Continuous mutations over time have resulted in extensive changes in HA proteins, resulting in all classified influenza strains in two phylogenetic-mediate major groups and six clades: Group 1 [H1 clade (H1, H2, H5 and H6); H9 clade (H8, H9 and H12); H11 clade (H11, H13, H16; and Bat Has clade (H17, and H18)] and Group 2 [(H3 clade (H3, H4 and H14) and H7 clade (H7, H10 and H15)]^10^. The HA1 subunit is used as a major factor in inducing immune responses in traditional vaccines; however, the presence of continuous mutations due to immune pressure in the HA1 subunit leads to a lack of cross-immune protection in the use of traditional vaccines for new strains^11,12^. In contrast, the HA2 subunit is known to be conserved in various strains of influenza as stem-located, furthermore, the HA2 subunit provides the neutralizing and protective antibodies against conformational epitopes and linear epitopes^13,14^. HA also mediates binding to sialic acid receptors and induces entry into host cells. HA, as the main influenza antigen is a key factor in the host-induced immune response^15^. NA is one of the other major envelope proteins. It has been shown that NA can secrete sialic acid in the form of enzymatic activity, thereby helping to release the virus^16^. Studies have shown that several mutations that alter the amino acid profile of HA can increase the binding affinity of HA to the sialic receptor in humans, thereby making the virus more susceptible to infection in humans^17–19^.

Using vaccination as a strategy to combat infections with potential epidemic and pandemic potential has led to significant advances in the combat against many of these infectious diseases, such as influenza^20^. Outbreaks appear to be exacerbated by influenza H7N9 strains, and the importance of rapid and effective influenza vaccines over existing vaccines^21^. To date, several vaccines have been developed against H7N9^22–25^. Subunit vaccines are very safe, specific, and rarely have post-vaccination side effects. Today, with the advancement of molecular techniques, the production of recombinant proteins has been made possible. This may help to produce a chimeric vaccine containing several virulence factors pathogenic because multicomponent subunit vaccines can elicit a more protective and efficient immune response than a single component vaccine^26^. The design and evaluation of immunogenicity of a candidate subunit vaccine are performed experimentally using laboratory techniques that are often time-consuming, costly and of low success rates^27^. Using bioinformatics software is a very viable and justifiable solution to help design and develop drugs and vaccines. Nowadays, in comparison with laboratory methods using predictable and reliable virtual methods for predicting B and T cell epitopes and MHC stimulation reduces the cost, time and risk of experiments. These methods have the following criteria: applicable to each host, the release of immunity at different sites of infection across different methods of application, and protection against different influenza^28^.

Due to the key role of HA and NA in host attachment and pathogenicity and since HA and NA are considered to the determination of the Influenza subtype, we used them as major antigens for designing of H7N9 influenza vaccine. Besides, HA and NA proteins were targeted to induce strong T cell responses specific for Cytotoxic T lymphocytes (CTL) and Helper T lymphocytes (HTL). Here, we report an effective insilico design strategy for making a recombinant vaccine. For this purpose, we analyzed the A/Shanghai/2/2013 influenza H7N9 strain sequence isolated from China. Suitable CTLs and HTLs epitopes, with the ability to elicit B cell lymphocyte (BCL) responses against HA and NA proteins, were selected, and the vaccine construct with appropriate linkers and two adjuvants were designed to increase the induction of cellular immunity.

## Results

### Phylogenetic evolution and Protein sequences retrieval

**Figure 1:**
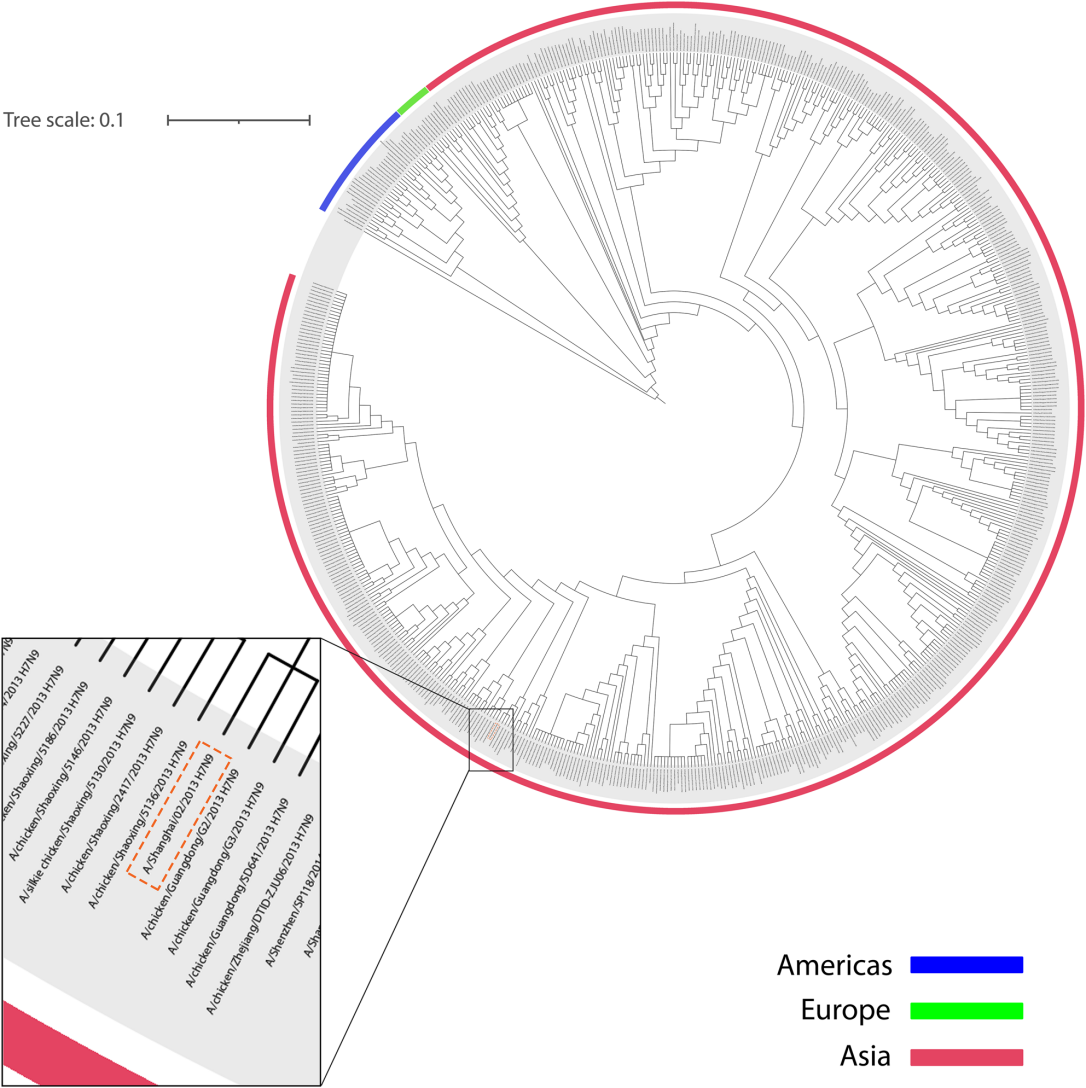
HA circular phylogenetic tree. The amino acid sequence of HA (A/Shanghai/2/2013 H7N9) was evaluated with other 642 (H7N9) HA amino acid-based on NCBI Influenza Virus Resource. The tree scale was considered equal to 0.1. All H7N9 HA proteins are related to human-derived, avian-derived and environment-derived. Spread H7N9 HA in the Americas, Europe and Asia are shown in blue, green and pink line, respectively. HA protein of A/Shanghai/2/2013 H7N9 has been magnified and specified in an orange dashed-line box.

**Figure 2:**
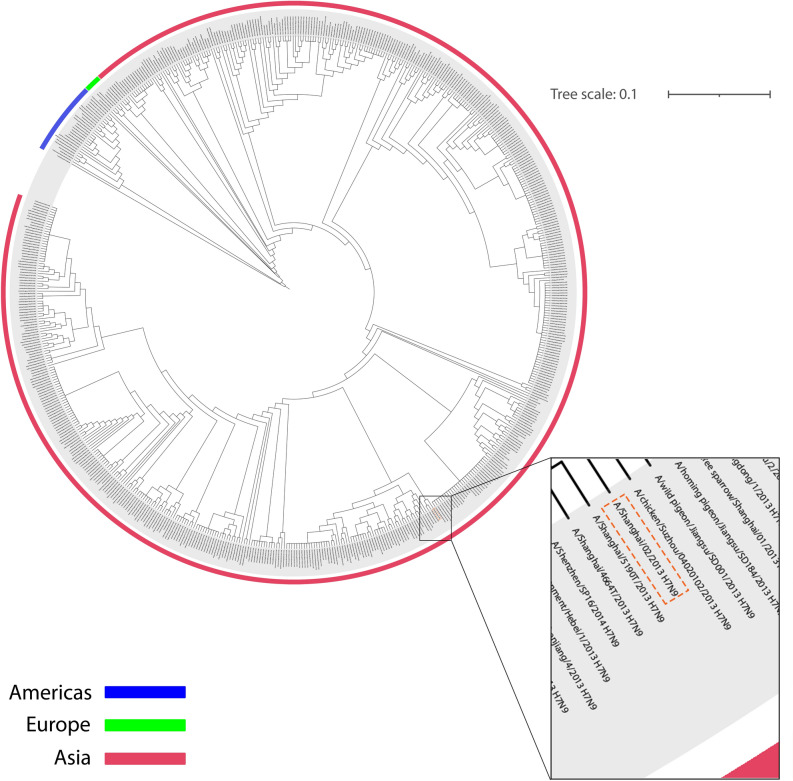
NA circular phylogenetic tree. The amino acid sequence of NA (A/Shanghai/2/2013 H7N9) was evaluated with other 620 (H7N9) NA amino acid-based on NCBI Influenza Virus Resource. The tree scale was considered equal to 0.1. All H7N9 NA proteins are related to human-derived, avian-derived and environment-derived. Spread H7N9 HA in the Americas, Europe and Asia are shown in blue, green and pink line, respectively. NA protein of A/Shanghai/2/2013 H7N9 has been magnified and specified in an orange dashed-line box.

Evolution relationships of NA and HA antigens selected from A/Shanghai/2/2013 show that they belong to the Eurasian strain of avian influenza viruses and are closely related to H7N9 subtypes spread to Europe and the United States. HA antigen of A/Shanghai/2/2013 can be seen in a common clade with avian-derived H7N9 (Fig. 1). Besides, HA shows a closer relationship with the 2013 and 2014 subtypes. But it still has a common ancestor with other H7N9s. In the same vein, NA antigen is also found in a clade with several human-derived H7N9s and avian-derived H7N9s (Fig. 2).

**Figure 3:**
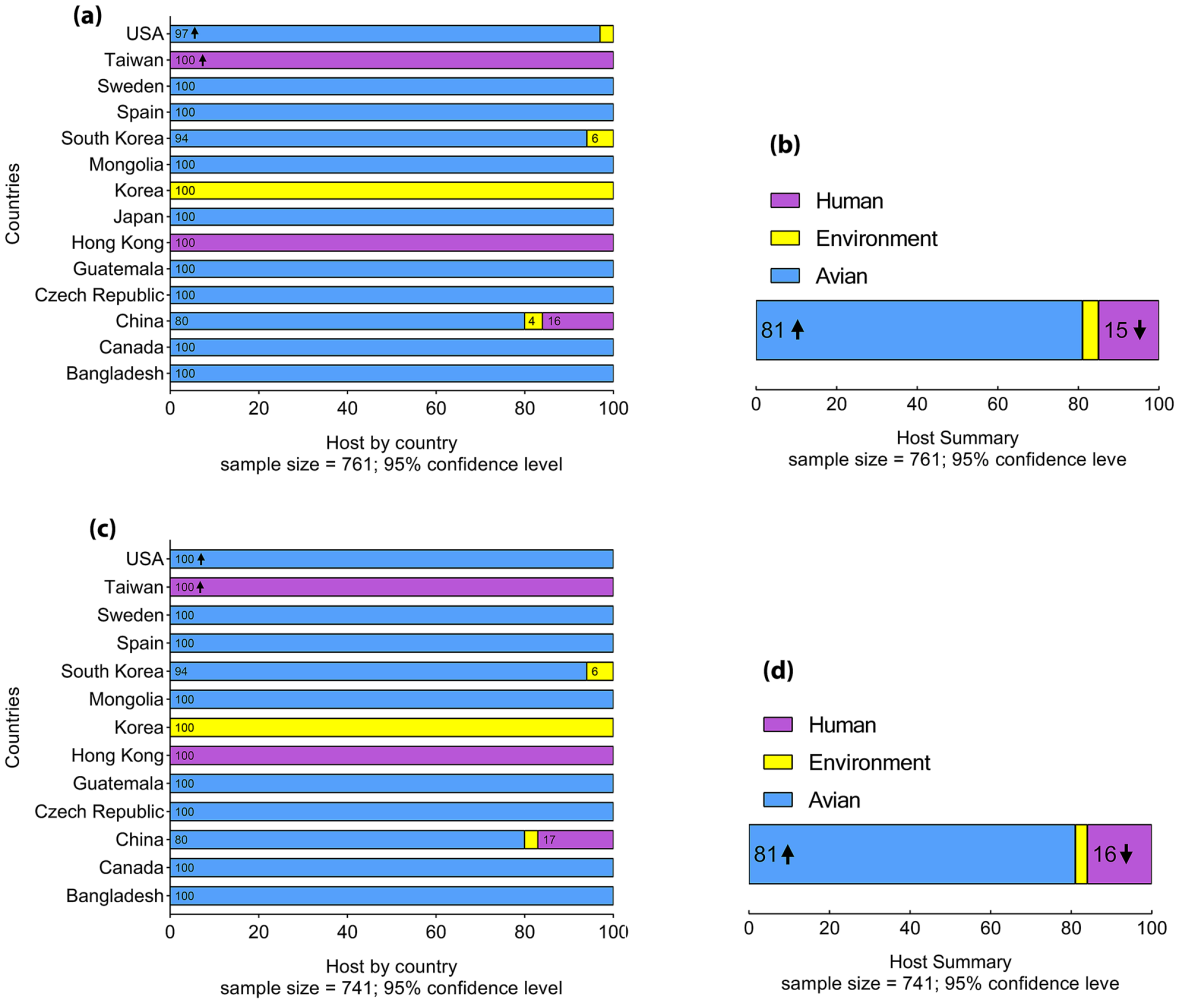
Chart frequency of HA and NA proteins from H7N9 Influenza. Frequency of H7N9 influenza HA protein, separately by country (**a**) and as a whole (**b**). Frequency of H7N9 influenza NA protein, separately by country (**c**) and as a whole (**d**). Human-derived, environment-derived and avian-derived H7N9 influenza (based on HA and NA protein) are shown in purple, yellow and blue, respectively.

HA and NA proteins of H7N9 influenza were investigated through categorized population frequently and as shown in Fig. 3a and c, it has spread in three continents of Asia, Europe and America. HA of H7N9 influenza is 100% avian-derived in Europe and Canada, while 3% environment-derived of H7N9 influenza was observed in the USA. H7N9 influenza was 16% human-derived in china, as well as 80% and 4% avian-derived and environment-derived, respectively. NA protein showed 100% avian- derived in both Europe and America. Similar to HA protein, NA protein in China was 80% avian-derived while 17% was human-derived.

It was clarified that more than 81% to been avian-derived among 761 HA proteins of H7N9 influenza, while less than 15% were human-derived and the rest were environment-derived (Fig. 3b). Also, 741 NA proteins of H7N9 influenza showed similar percent so that more than 81% were related to avian-derived and less than 16% were estimated to human-derived (Fig. 3d).

### Linear and conformational B cell epitopes prediction

B cell epitopes are an important component of the H7N9vac because of their ability to induce humoral immunity. Both linear and discontinuous B cell epitopes in the final construct were predicted by the BepiPred 2.0 and iBCE-EL servers, respectively. A total of 27 linear B cell epitopes were extracted from the final construct and shown in Table 1. Conformational B cell epitopes from HA and NA are summarized in supplementary Table S1. Each of the 8 predicted scores for conformational B cell epitopes was visualized in supplementary Fig. S1.

**Table 1:** Liner B-cell epitopes predicted from HA and NA antigens.

Antigen	Residues	Vaxigen	iBCE-EL
**HA**			
49–72	ETVERTNIPRICSKGKRTVDLGQC	0.6722	BCE
83–90	QCDQFLEF	0.4864	BCE
99–115	REGSDVCYPGKFVNEEA	0.4214	BCE
117–152	RQILRESGGIDKEAMGFTYSGIRTNGATSACRRSG**S**	0.0803	BCE
164–182	NTDNAAFPQMTKSYKNTRK	0.3247	BCE
196–207	STAEQTKLYGSG	− 0.0864	BCE
217–238	NYQQSFVPSPGARPQVNG**LSGR**	0.6784	BCE
274–285	**MGI**QSGVQVDAN	0.6137	BCE
292–311	HSGGTIISN**LPFQNIDSRAV**	0.5661	BCE
322–339	**SLLLATGMKNV**PEIPKGR	0.3190	BCE
351–382	NGWEGLIDGWYGFRHQNAQGEGTAADYKSTQS	0.4809	BCE
399–425	**NQQFELIDNEF**NEVEKQIGNVINWTRD	0.6330	Non-BCE
445–460	**HTIDL**ADSEMDKLYER	0.5966	BCE
467–474	ENAEEDGT	0.0449	BCE
491–513	IRNNTYDHSKYREEAMQNRIQID	0.2442	BCE
**NA**			
40–55	GCNCSHSQPETTNTSQ	0.3353	BCE
71–87	**QMEERT**SRNFNNLTKGL	0.7129	BCE
134–151	TTIRGKHSN**GTIHDRSQY**	1.0203	BCE
160–167	SSPPT**VYN**	0.1887	BCE
207–221	RPVAEINTWARNILR	− 0.8287	Non-BCE
242–248	SATGPAD	0.4148	BCE
257–268	GKILKWESLTGT	0.4550	Non-BCE
319–343	LTDNPRPNDPNIGKCNDPYPGNNNN	0.5122	BCE
359–370	GRT**ISTASRSGY**	0.7779	BCE
377–388	NALTDDRSKPIQ	0.7779	BCE
394–401	LNADWSGY	0.7684	BCE
448–461	EFLGQWNWPDGAKI	0.2604	Non-BCE

Overlapped liner B cell epitopes with conformational B-cell epitopes are shown in bold characters.

### Selection of CTL epitopes prediction

The NetMHC4 server was used for HA and NA MHC class I prediction. MHC-I epitopes were selected based on the highest immunogenicity and antigenicity. Furthermore, MHC-I epitopes were considered with a strong binding score based on the NetMHC4 server rating. Selected MHC-I epitopes that had overlapped with each other were merged and finally 13 MHC I epitopes, including 8 HA epitopes and 5 NA epitopes were utilized at influenza construct (Table 2). All predicted CTL epitopes (binding to MHC-I) are included in Supplementary Table S2.

**Table 2:** CLT epitopes prediction by NetMHC4 server. HA and NA epitopes were selected based on MHC-I HLAs binding affinity (nM), %rank and antigenicity rate.

Pos	HLA	Peptide	Rank^a^	Antigenicity	TAP transport	Proteasomal C-terminal cleavage	Comb	Rank^b^	Conservancy
HA									
151–159	HLA-A3215	SSFYAEMKW	0.08	1.0372	1.06000	0.94850	0.48591	2.0	97.24%
151–159	HLA-B5701	SSFYAEMKW	0.01	1.0372	1.06000	0.94850	0.88591	0.05	97.24%
151–159	HLA-B5802	SSFYAEMKW	0.06	1.0372	1.06000	0.94850	0.58491	0.15	97.24%
234- 242	HLA-B5801	**LSGR**I**DFHW**	0.01	2.2921	0.73600	0.60346	1.01918	0.05	82.02%
236–244	HLA-B2705	**GRIDFHWLM**	0.25	2.2522	0.45500	0.97351	0.89141	0.05	89.37%
251–259	HLA-A2603	**VTFS**FNGAF	0.05	1.0613	2.69700	0.69959	0.50983	1.50	91.60%
267–275	HLA-A0203	FLRGKS**MGI**	0.04	1.0137	0.56200	0.86838	1.03243	0.20	90.16%
267–275	HLA-A0250	FLRGKSMGI	0.08	1.0137	0.56200	0.86838	0.90743	1.50	90.16%
300–308	HLA-B8301	**LPFQNIDSR**	0.09	1.1190	1.43700	0.89025	0.42223	10.0	85.70%
308–316	HLA-A3002	**RAV**GKCPRY	0.17	1.0469	3.35300	0.90995	0.83056	0.80	92.52%
440–448	HLA-B4002	MENQ**HTIDL**	0.20	1.0044	0.92600	0.94215	0.89213	0.20	98.29%
440–448	HLA-B4402	MENQ**HTIDL**	0.15	1.0044	0.92600	0.94215	0.64313	0.20	98.29%
526–534	HLA-A2902	**WFSFGASCF**	0.40	1.7382	2.71900	0.12658	0.51046	4.00	97.90%
526–534	HLA-C0401	**WFSFGASCF**	0.01	1.7382	2.71900	0.12658	0.14546	50.0	97.90%
527–535	HLA-B4013	**FSFGASCFI**	0.09	1.3232	0.53600	0.28604	0.33576	7.00	91.08%
527–535	HLA-C0303	**FSFGASCFI**	0.07	1.3232	0.53600	0.28604	0.46876	4.00	91.08%
NA									
24–32	HLA-A0203	**GMANLGLNI**	0.40	1.2411	0.50300	0.76733	0.81222	1.50	0.40%
142–150	HLA-A6601	**GTIHDRSQY**	0.15	1.5360	2.93400	0.72211	0.46883	1.50	95.96%
164–172	HLA-A2402	**VYN**SRVECI	0.25	1.4709	0.77700	0.44486	0.56952	1.50	93.40%
361–369	HLA-B1517	**ISTASRSGY**	0.09	1.0412	2.96200	0.68178	1.03945	0.40	92.86%

TAP transport/Proteasomal C-terminal cleavage potential selected epitopes were evaluated and degree of conservation was considered for all selected epitopes. Overlapped CLT epitopes are shown in underlined characters as well as CLT epitopes overlapped with HTL epitopes are shown in bold characters.

^a^Rank Threshold for Strong binding peptides is equal to < 0.5 and for Weak binding peptides is equal to < 2.0.

^b^Rank Threshold for epitope identification is equal to < 1.0.

### TAP transport/proteasomal cleavage

Due to the importance of TAP transport and Proteasomal cleavage in the antigen presentation pathway binding affinity, HA and NA antigens were examined via the NetCTL1.2 servers. The scores associated with tap transport and Proteasomal cleavage for each of the CTL epitopes shown in Table 2 are shown. The most immunogenic CTL epitopes were used for this section.

### Selection of HTL epitopes prediction

HA and NA antigens were submitted in NetMHCII—2.3 to predict the binding of peptides to MHC class II alleles. All MHC class II epitopes were analyzed and epitopes were selected which show the highest immunogenicity and antigenicity with the stronger binding score. MHC class II epitopes that had showing overlap were considered independent epitopes. All MHC class II epitopes were analyzed based on inducing of IFN-γ and were included in the final construct. A total of 5 MHC class II epitopes including 3 HA and 2 NA epitopes were selected for the construction of the H7N9-Vaccine (H7N9vac) (Table 3). All predicted HTL epitopes (binding to MHC-II) are included in Supplementary Table S3.

**Table 3:** HTL epitopes prediction by NetMHCII 2.3 server.

Allele	Pos	Peptide	Rank^a^	Antigenicity	IFNepitope SVM^b^	Conservancy
**HA**						
DRB1_0101	240–254	**DFHWLML**NPND**TVTF**	0.90	1.4982	POSITIVE	89.24%
DRB1_1302	241–256	**FHWLML**NPND**TVTFS**	1.50	1.1847	POSITIVE	88.85%
DRB3_0202	241–256	**FHWLML**NPND**TVTFS**	0.60	1.1847	POSITIVE	88.85%
DRB3_0301	241–256	**FHWLML**NPND**TVTFS**	0.70	1.1847	POSITIVE	88.85%
DRB5_0101	318–332	VKQR**SLLLATGMKNV**	2.50	1.0222	POSITIVE	69.16%
DPA10103-DPB10301	318–332	VKQR**SLLLATGMKNV**	1.00	1.0222	POSITIVE	69.16%
DPA10201-DPB10501	318–332	VKQR**SLLLATGMKNV**	0.40	1.0222	POSITIVE	69.16%
DQA10501-DQB10201	395–409	IEKT**NQQFELIDNEF**	1.40	1.1539	POSITIVE	79.00%
DPA10103-DPB10401	525–539	IL**WFSFGASCFI**LLA	0.50	1.0849	POSITIVE	90.29%
DRB1_0403	539–553	AIVMGLVFICVKNGN	0.06	1.3687	POSITIVE	83.99%
**NA**						
DRB1_0404	20–34	IAVLI**GMANLGLNI**G	1.50	1.4039	POSITIVE	0.40%
DRB1_0402	21–35	AVLI**GMANLGLNI**GL	1.70	1.4582	POSITIVE	0.40%
DRB1_0403	21–35	AVLI**GMANLGLNI**GL	1.60	1.4582	POSITIVE	0.40%
DRB4_0103	21–35	AVLI**GMANLGLNI**GL	9.50	1.4582	POSITIVE	0.40%
DQA10102-DQB10501	21–35	AVLI**GMANLGLNI**GL	1.30	1.4582	POSITIVE	0.40%
DRB1_1302	23–37	I**GMANLGLNI**GLHLK	1.60	1.9099	POSITIVE	0.40%
DRB1_0401	59–73	NNYYNETNITNI**QME**	1.20	1.0803	POSITIVE	79.65%
DQA10401-DQB10402	61–75	YYNETNITNI**QMEER**	1.20	1.4272	POSITIVE	78.17%
DQA10301-DQB10302	62–76	YNETNITNI**QMEERT**	1.90	1.4338	POSITIVE	78.03%
DRB4_0101	297–311	RPVIQIDPVAMTHTS	1.30	1.0194	POSITIVE	92.18%
DRB3_0101	298–312	PVIQIDPVAMTHTSQ	1.80	1.1009	POSITIVE	92.18%

HA and NA epitopes were selected based on MHC-II HLAs binding affinity (nM), %rank and antigenicity rate. All epitopes were evaluated based SVM^a^ method as IFN-γ inducer potential and degree of conservation were considered for all selected epitopes. Overlapped HLT epitopes are shown in underlined characters as well as HTL epitopes overlapped with CTL epitopes are shown in bold characters.

^a^Rank Threshold for Strong binding peptides is equal to < 0.5 and for Weak binding peptides is equal to < 2.0.

^b^Motif and SVM hybrid/IFN-gamma versus other cytokines.

### Population coverage

**Figure 4:**
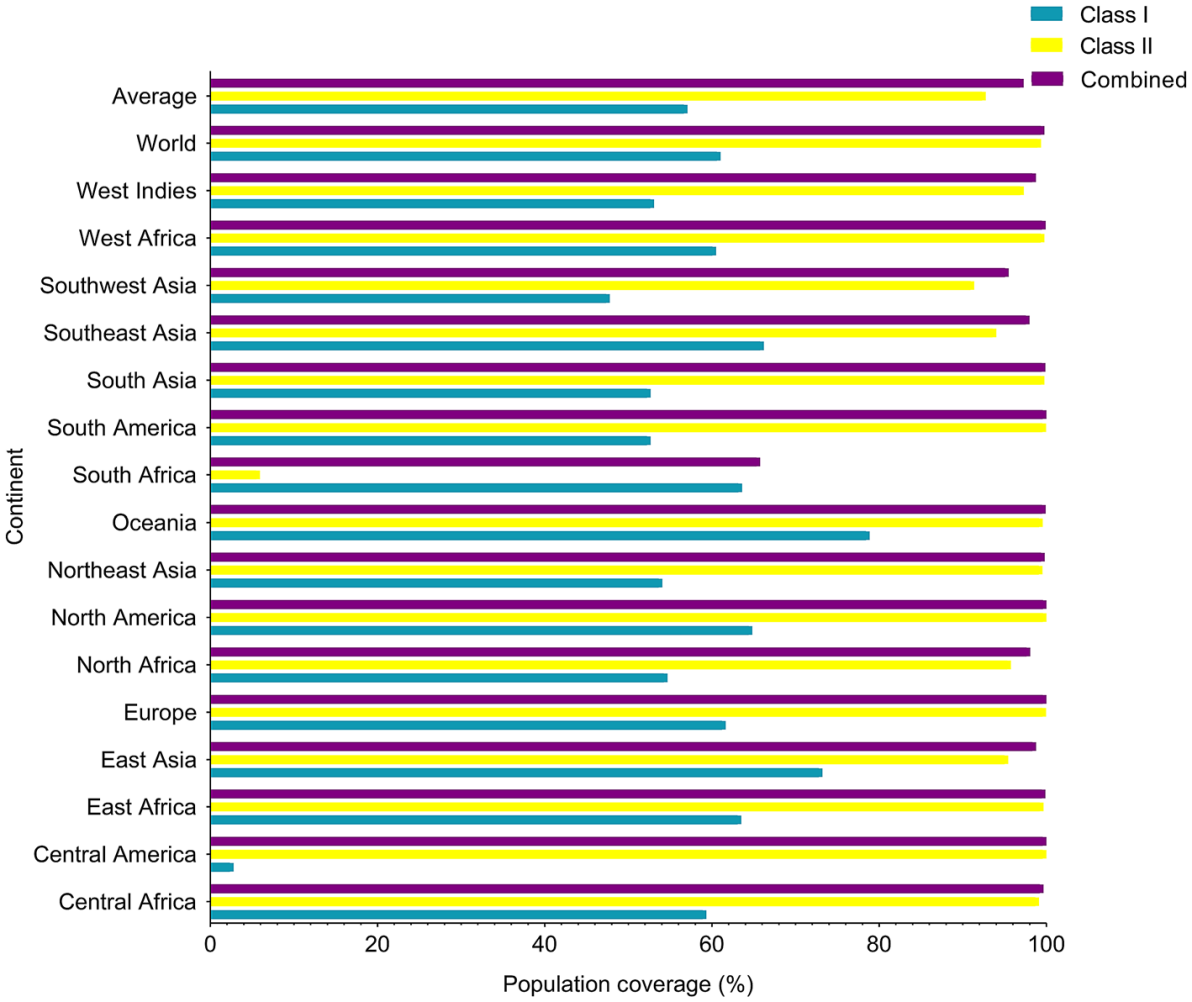
Population coverage (%) related to selected epitopes HLA binding alleles. A worldwide and an average percentage of population coverage is considered for all selected epitopes. MHC class I and MHC class II and combined MHC class I and MHC class II are shown in blue, yellow and purple color, respectively.

IEDB population coverage tool in http://tools.iedb.org/population was used to determine the coverage rate of the population for any selected epitope. As can be seen in Fig. 4, the highest population coverage is observed in combined Class I and II, given that the HA and NA antigens associated with the A Shanghai/2/2013 antigen originate from China in Asia. Detailed Class I, Class II and combined Class I and II epitopes population coverage related to continents are summarized in Supplementary Table S4 and Supplementary Fig. S2.

### Construction design and physicochemical properties

**Figure 5:**
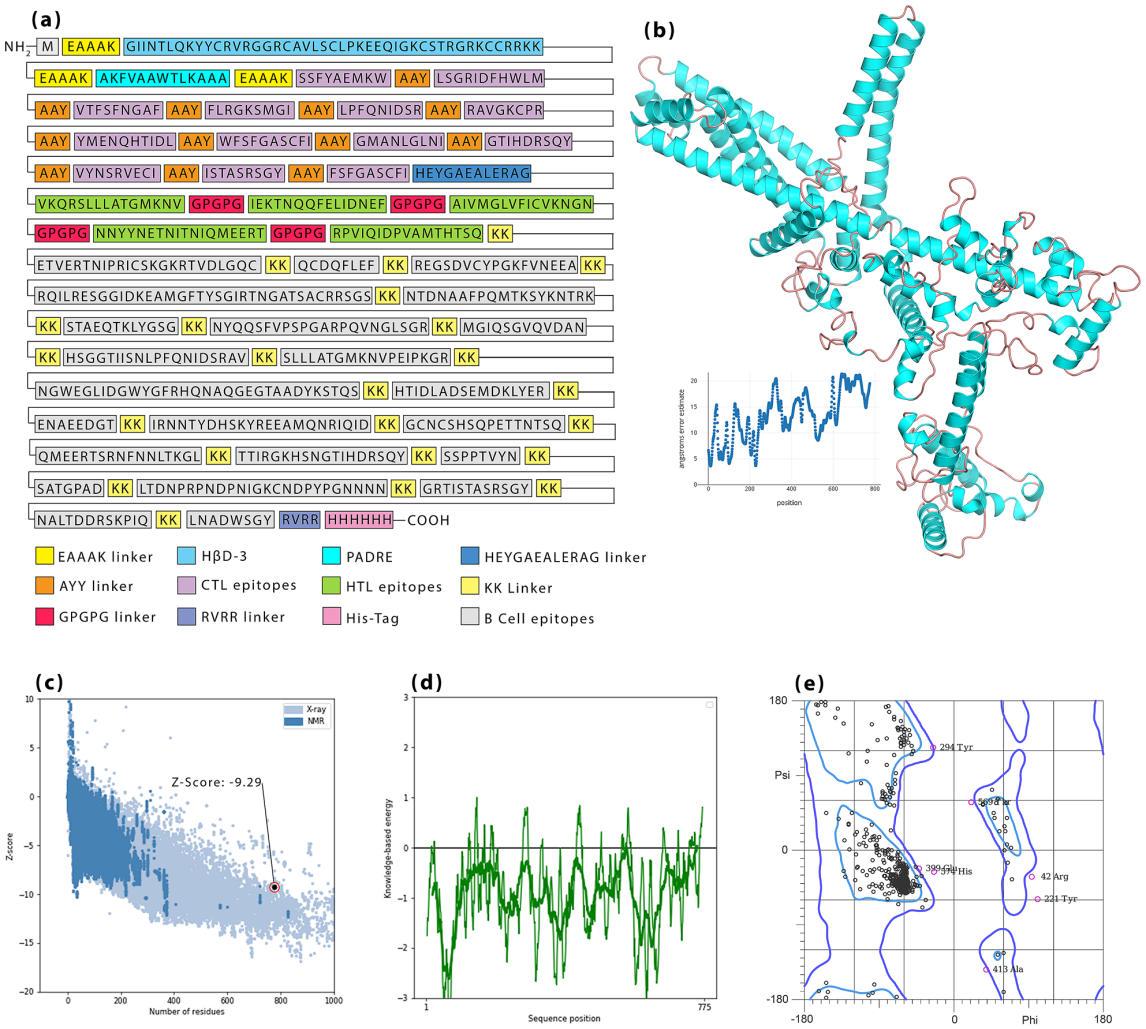
(**a**) Representation of H7N9vac construct schematic including linkers, adjuvants, CTL and HTL epitopes. EAAAK linkers connect the adjuvants, AAY linkers connect CTL epitopes, HEYGAEALERAG linker connects last CTL epitope to first HTL epitope, GPGPG linkers connect HTL epitope and RVRR linker connects Histidine-Tag to final construct. (**b**) Modeled 3D structure of H7N9vac. (**c**) Z-Score validation, (**d**) Local model quality and (**e**) Ramachandran plot related to vaccine 3D structure after refinement.

Final selected epitopes were linked by linkers so that AAY linkers were selected for linking of MHC-I epitopes as well as GPGPG linkers for linking of MHC-II epitopes. HβD-3 and PADRE sequence as adjuvants were linked by EAAAK linkers in the N-terminal site. The last MHC-I epitope was linked to the first MHC-II epitope via the HEYGAEALERAG linker. KK linkers also were used to bind B-cell epitopes. A “Histidine Tag” was considered in the C-terminal and linked to the final construct through the RVRR linker (Fig. 5a).

Obtained results from the ProtParam server demonstrated H7N9vac amino acid sequence (777 amino acid) will translate into a protein with a molecular weight of ~ 86.14 kDa. Theoretical pI and instability index (II) were estimated equal to 9.80 and 34.65, respectively, that it grouped Influenza vaccine in stable proteins. Furthermore, the influenza vaccine is expected to have a half-life of 10–30 h in the various prokaryotic and eukaryotic hosts. The grand average of hydropathicity (GRAVY) was computed to be − 0.834, as well as an Aliphatic index of 56.26 (Table 4). The predicted secondary structure of the final construct is presented in Supplementary Fig. S3.

**Table 4:** Physicochemical and immunological properties of the final construct.

Indicators	MW	pI	II	AI	GRAVY	Antigenicity	Allergenicity	Toxicity
Final construct	~ 86.38 kDa	9.80	34.65	56.26	− 0.834	0.6306	Non-allergen	Non-toxic

The possibility of disulfide bonds was investigated by the DiANNA server and it was found that the disulfide bonds have been stabilized between ^17^Cys-Cys^168^, ^24^Cys-Cys^630^, ^29^Cys-Cys^46^, ^39^Cys-Cys^359^, ^47^Cys-Cys^374^, ^142^Cys-Cys^280^, ^205^Cys-Cys^628^, ^343^Cys-Cys^417^ and ^355^Cys-Cys^717^ amino acids.

### Modeling of 3D construct, refinement and validation

RoseTTAFold server was used to model H7N9vac 3D structure (Fig. 5b). Next step, the achieved H7N9vac 3D structure submitted to the GalaxyRefine server. The predicted model validation was performed by online software PROCHECK, PROSA. As shown in Fig. 5c, the Z-Score point was set to − 9.29 after refinement. The Z-Score point is used to validate the modeled protein based on the NMR or X-ray methods. The NMR method is used for the crystallography of proteins with fewer than 200 amino acids and the X-ray method for proteins with more than 200 amino acids. If the Z-Score dot in the PROSA graph is located on the (blue–blue) NMR and (pale-blue) X-ray regions, the simulation accuracy is the highest and the simulated model has the lowest error rate with the highest confidence. Local model quality related to structure is shown in Fig. 5d.

Model analysis of the predicted model after drawing the Ramachandran graph revealed that 92.4% (714/773) of all residues were in favored (98%) regions. Overall, approximately 97.8% (756/773) of all residues were in allowed (> 99.8%) regions. In the Ramachandran graph, amino acids are grouped according to the angles of phi and psi (Fig. 5e).

### Insilico cloning and vaccine optimization

**Figure 6:**
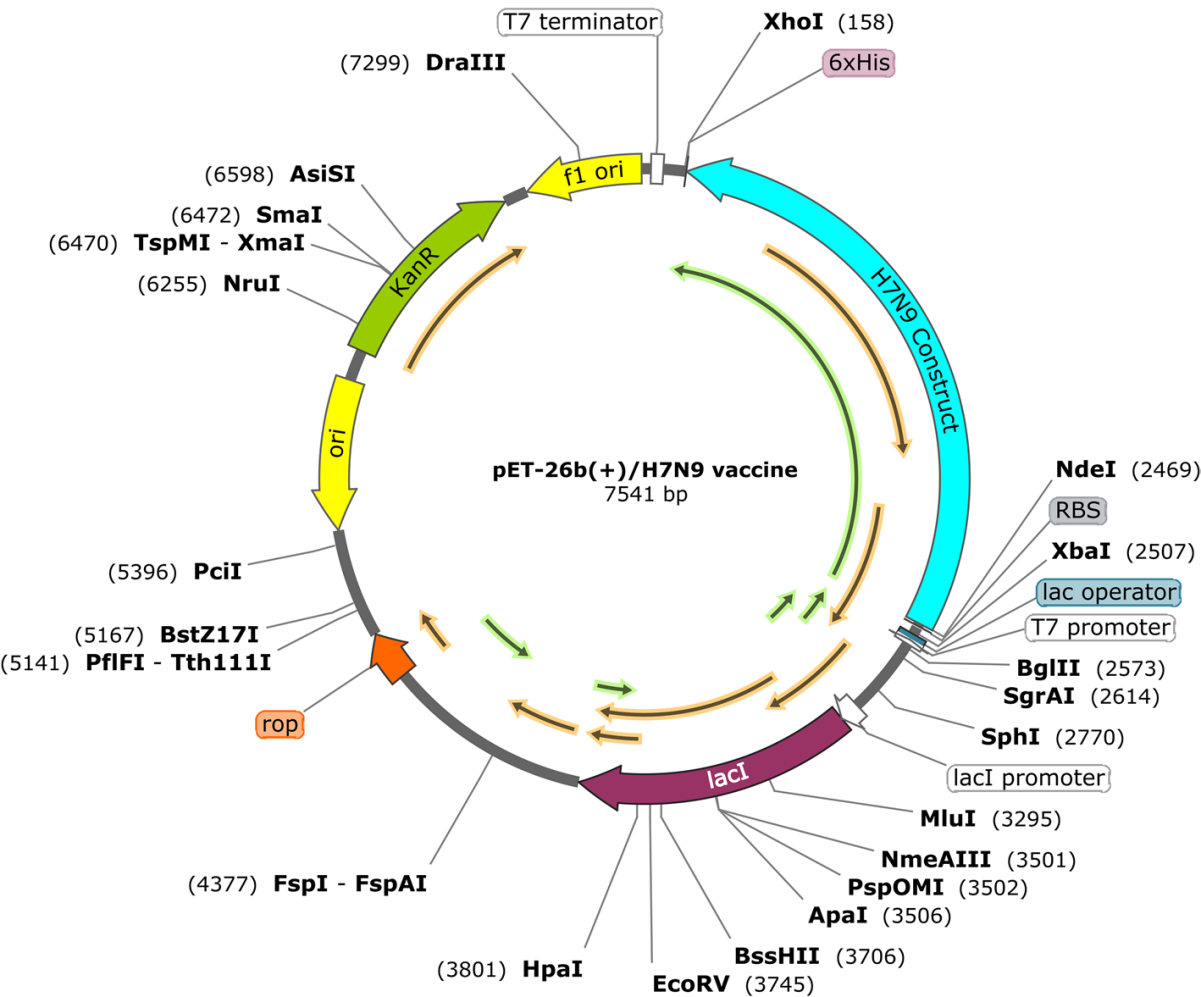
Insilico cloning of H7N9 nucleotide sequence insertion in pET26b(+) by SnapGene. The H7N9 nucleotide sequence is shown as the blue line. *Nde*I and *Xho*I restriction enzymes are represented in the N-terminal and C-terminal of the H7N9 construct, respectively. Considered His-Tag is shown as a purple box in C-terminal pET26b(+).

Codon Adaptation Tool (JCAT) was used for the optimization of nucleotide sequences of the final construct. *E. coli* K12 was selected as a host expression organism. Also, we adjust the properties of JCAT to avoid rho-independent transcription terminators, avoid prokaryotic ribosome binding sites and avoid cleavage sites of restriction enzymes. Codon Adaptation Index-Value (CAI -Value) and GC-Content of the improved Influenza construct with 2310 nucleotide sequence (without His-Tag) were 1.0 and 54.20, respectively, which confirms the probability of appropriate protein expression. *Nde* I and *Xho* I restriction enzymes was added to the N-terminal and C-terminal of nucleotide sequence, respectively, so that the PelB sequence is removed from the final structure for achieving an intracellular expression. Furthermore, a stop codon was considered after His-Tag sequence. Finally, the construct was cloned in pET-26b(+) plasmid by SnapGene (Fig. 6).

Nucleotide sequence of H7N9-Vac along with Open reading frames of protein expression are included in Supplementary Table S5 and Supplementary Fig. S4, respectively.

### Protein–protein docking

**Figure 7:**
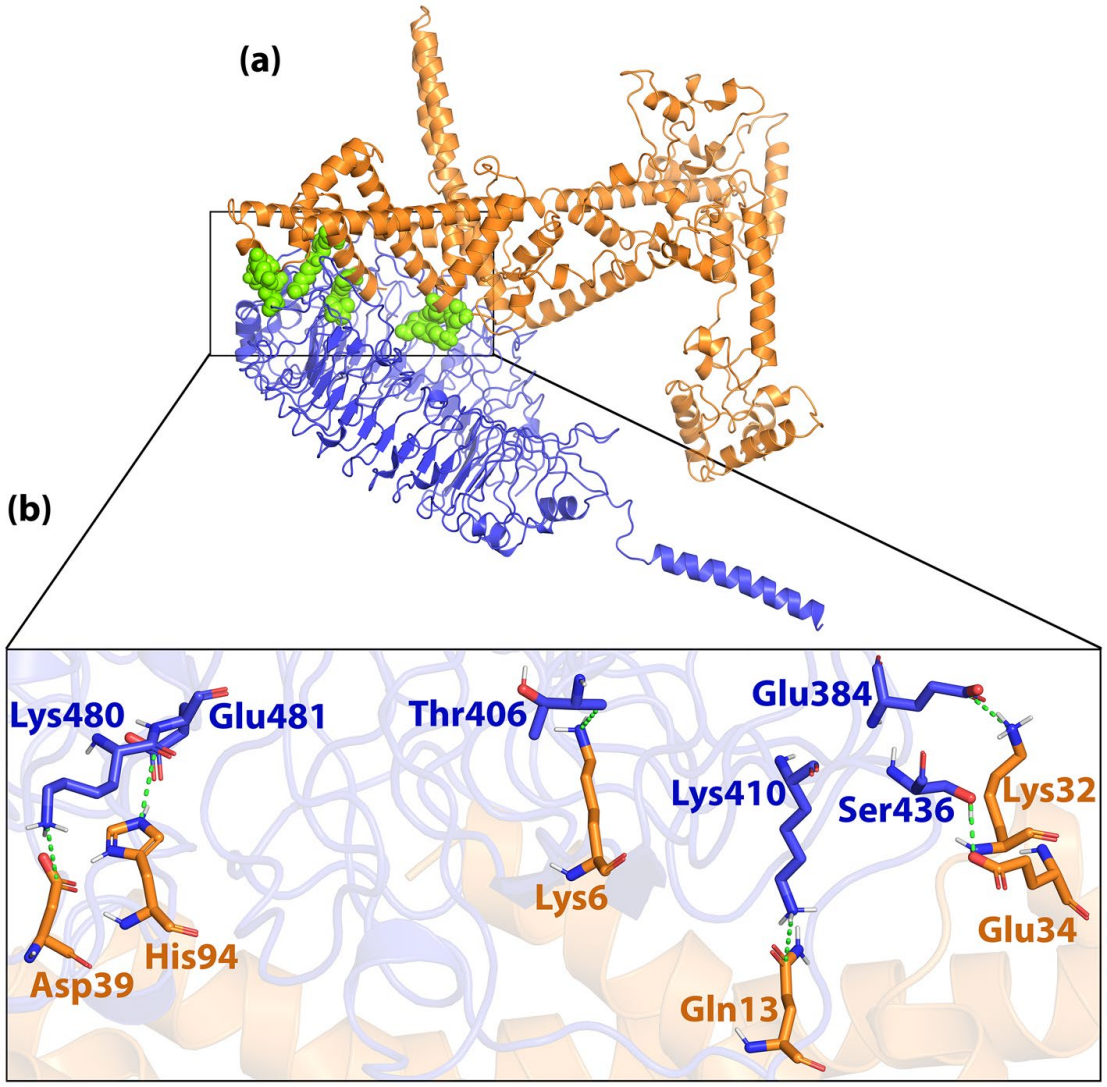
(**a**) Visualization of docking HADDOCK results for H7N9vac-TLR7 complex and (**b**) magnified residues interaction of H7N9vac-TLR7 complex. H7N9v structure is shown as the carton and sticks in copper color. TLR7 structure is shown as the carton and sticks in blue color. Hydrogen bonds are shown as the green dashed line.

**Figure 8:**
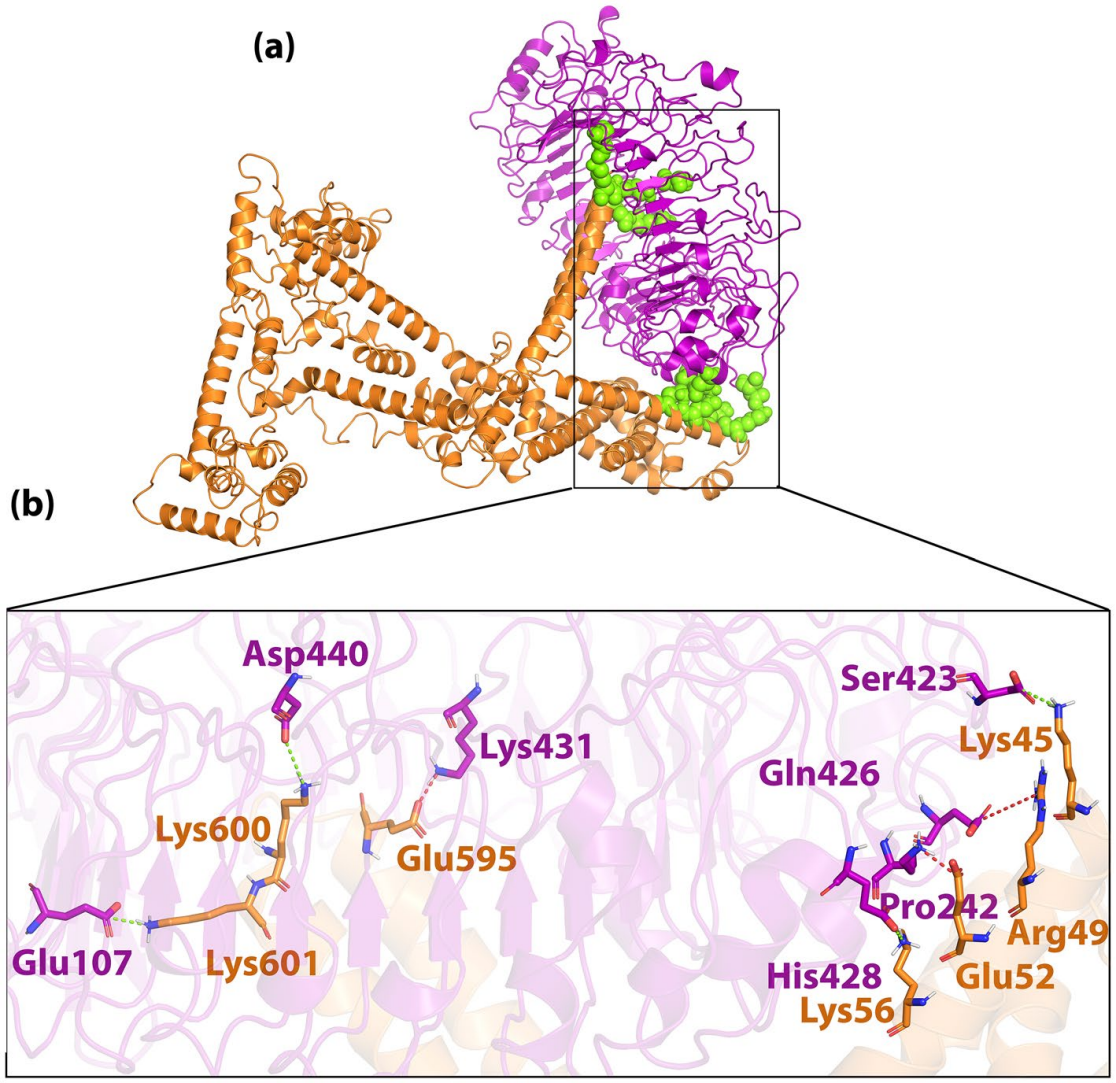
(**a**) Visualization of docking HADDOCK results for H7N9vac-TLR8 complex and (**b**) magnified residues interaction of H7N9vac-TLR8 complex. H7N9v structure is shown as the carton and sticks in copper color. TLR8 structure is shown as the carton and sticks in purple color. Hydrogen bonds are shown as the green dashed line and salt bridges are shown as the red dashed line.

Docking results showed H7N9vac can form interaction with TLR7, TLR8 and m826 antibody 3D structures. We selected and included two PADRE and HβD-3 sequences as the adjuvant in the N-terminal of the final construct. As shown in Figs. 7a and 8a, initial areas amino acids of the H7N9vac were involved with TLR7 and TLR8. Furthermore, the H7N9vac directly formed interaction with several amino acids of TLR7 and TLR8 binding sites.

**Figure 9:**
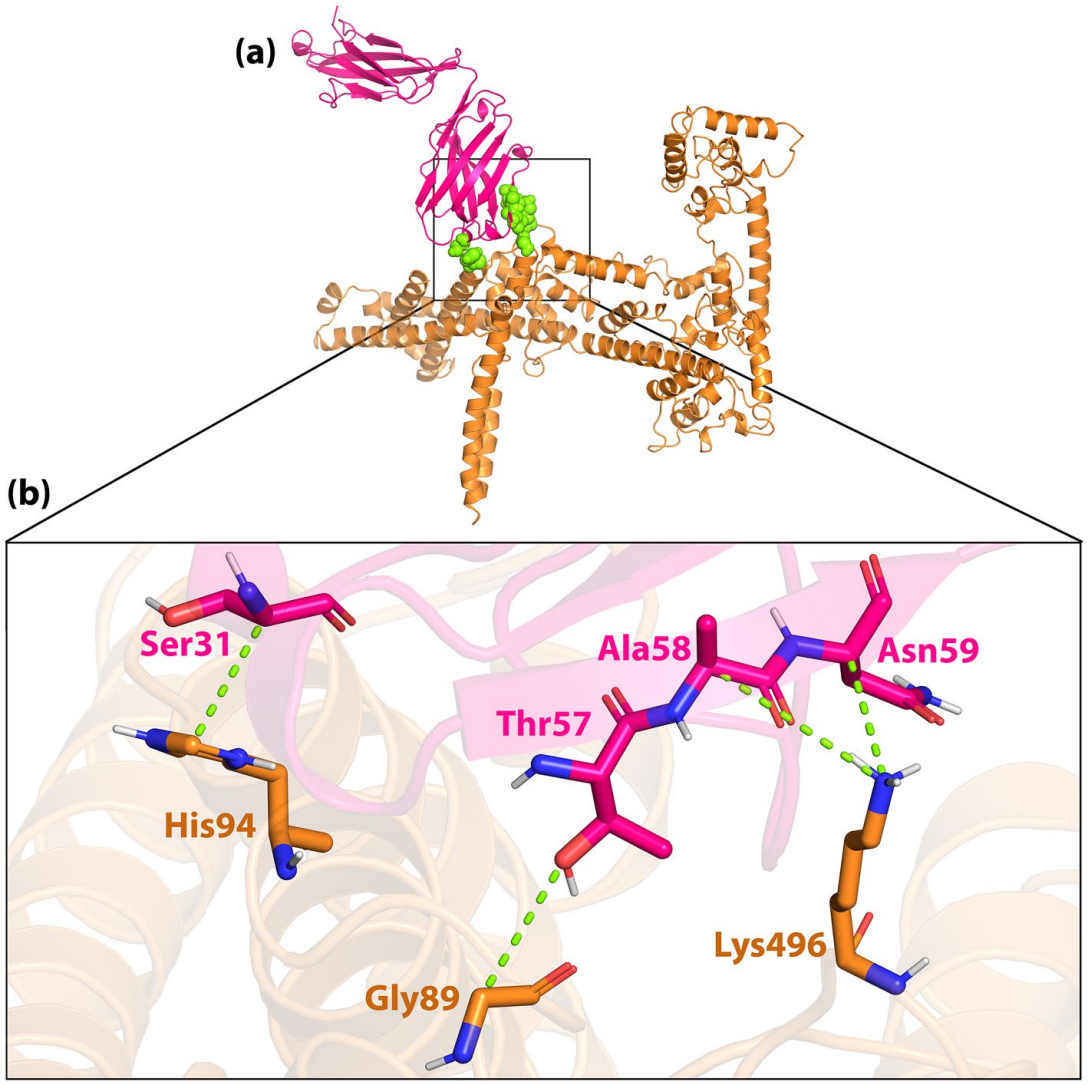
(**a**) Visualization of docking HADDOCK results for H7N9vac-m826 Heavy chain complex and (**b**) magnified residues interaction of H7N9vac-m826 Heavy chain complex. H7N9v structure is shown as the carton and sticks in copper color. m826 Heavy chain structure is shown as the carton and sticks in pink color. Hydrogen bonds are shown as the green dashed line.

**Figure 10:**
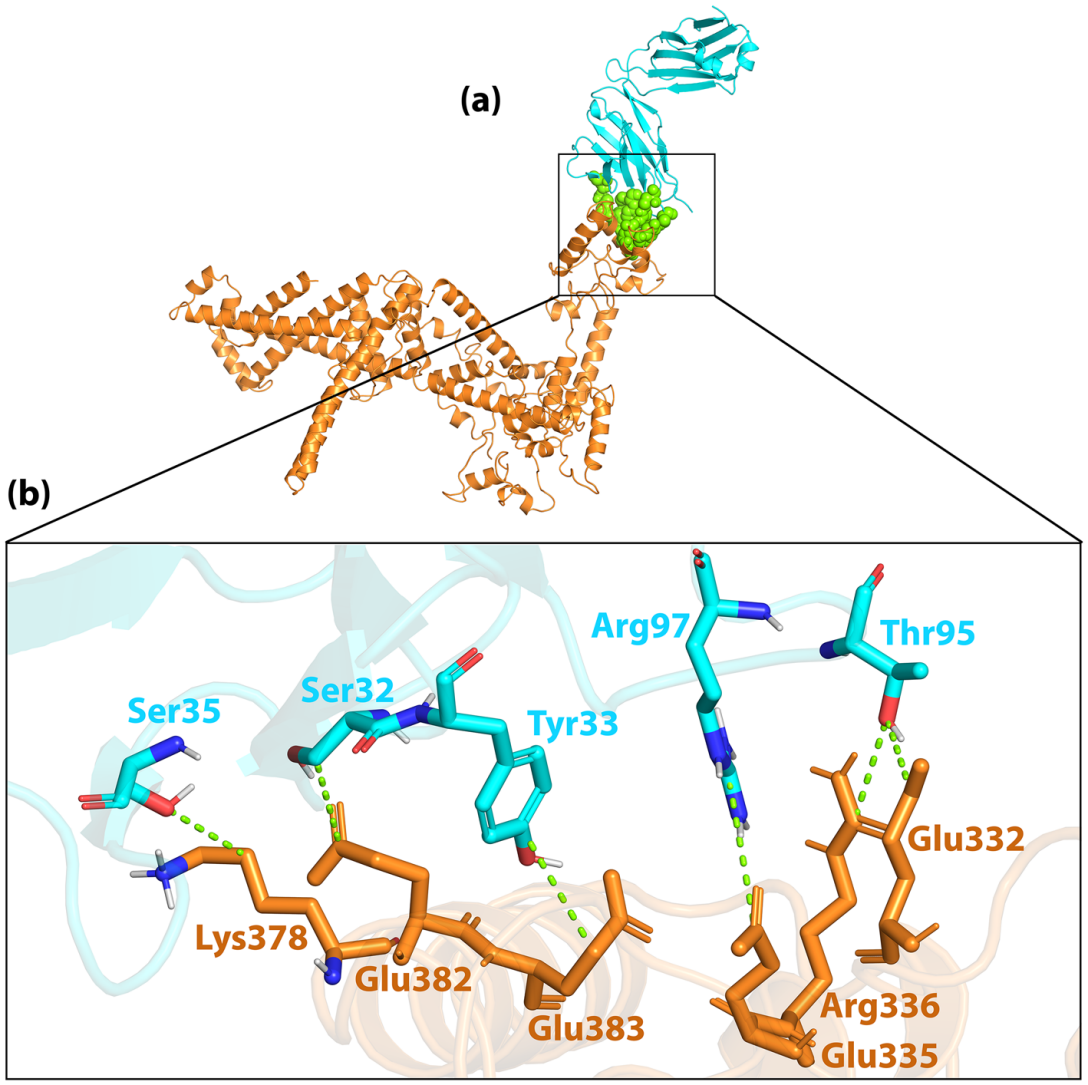
(**a**) Visualization of docking HADDOCK results for H7N9vac-m826 Light chain complex and (**b**) magnified residues interaction of H7N9vac-m826 Light chain complex. H7N9v structure is shown as the carton and sticks in copper color. TLR8 structure is shown as the carton and sticks in cyan color. Hydrogen bonds are shown as the green dashed line.

The H7N9vac was also able to interact with both the heavy and light chains of the m826 antibody (Figs. 9a and 10a). HCDR1, HCDR2 and HCDR3 loops from the heavy chain and LCDR1, LCDR2 and LCDR3 loops from the light chain were selected from the m826 antibody to bind to specific regions of the HA antigen in H7N9vac. As shown in Fig. 9a and b as well as Fig. 10a, and b, the interaction was established between the amino acids His94, Gly89 and Lys496 from H7N9vac with the amino acids Ser31, Thr57, Ala58 and Asn59 heavy chain m826 antibody. The amino acids Lys378, Glu382, Glu383, Glu335, Arg336 and Glu332 from H7N9vac also are interacted with the amino acids Ser35, Ser32, Tyr33, Arg97 and Thr95 from the m826 antibody light chain.

The H7N9vac’s structure and TLR7, TLR8 and m826 were submitted to the HADDOCK server for identifying the interaction reigns by protein–protein docking, respectively. The highest rankings for each complex were selected at the lowest intermolecular binding of the whole-molecular H7N9vac-Proteins complexes from the HADDOCK with the lowest mean RMSD (Table 5). As shown in Fig. 7b, the H7N9-vac interacted with TLR7 via 6 hydrogen bands. Besides, further investigations of HADDOCK server output show that electrostatic bonds also play an important role in the interactions generated in the H7N9vac-TLR7 complex. In this regard, in the H7N9vac-TLR8 complex, 3 hydrogen bonds and 4 salt bridges are responsible for creating interactions between the H7N9vac and TLR8, just as electrostatic bonds play a major role in the interactions of this complex (Fig. 8b).

**Table 5:** HADDOCK clusters results related to H7N9vac-TLR7, H7N9vac-TLR8, H7N9vac-m826 Heavy chain and H7N9vac-m826 Light chain docked complexes. The most reliable cluster was selected based HADDOCK score for each complex.

Complexes	H7N9vac-TLR7	H7N9vac-TLR8	H7N9vac-m826 Heavy chain	H7N9vac-m826 Light chain
HADDOCK score (kJ/mol)	− 77.9 ± 34.0	3.5 ± 4.0	− 54.3 ± 6.8	− 35.3 ± 1.9
Cluster size (nm)	6	13	53	101
RMSD from the overall lowest-energy structure (nm)	47.2 ± 0.5	7.3 ± 0.1	1.3 ± 1.0	14.3 ± 0.5
Van der Waals energy (kJ/mol)	− 98.6 ± 9.7	− 55.7 ± 3.3	− 48.5 ± 1.4	− 24.3 ± 2.4
Electrostatic energy (kJ/mol)	− 266.6 ± 81.2	− 523.3 ± 21.9	− 55.2 ± 15.1	− 161.6 ± 33.2
Desolvation energy (kJ/mol)	28.3 ± 16.0	83.8 ± 4.5	− 3.3 ± 3.4	13.1 ± 5.9
Restraints violation energy (kJ/mol)	457.3 ± 61.14	799.8 ± 58.89	86.0 ± 9.59	81.4 ± 26.44
Buried Surface Area	3348.5 ± 458.8	2584.1 ± 80.6	1134.8 ± 15.9	1049.7 ± 29.4
Z-Score	− 1.5	− 1	− 1	− 1.4

Regarding the binding of the vaccine to the m826 antibody, 4 hydrogen bonds in H7N9vac-m826 Heavy chain complex and 6 hydrogen bonds in H7N9vac-m826 Light chain complex were responsible for the interaction between the vaccine and the antibody, respectively (Figs. 9b and 10b). 2D interactions are summarized and visualized in Supplementary Fig. S5. Besides, Graphical HADDOCK results are presented in Supplementary Figs. S6, S7, S8 and S9.

### Molecular dynamic and MM/PBSA analysis

**Figure 11:**
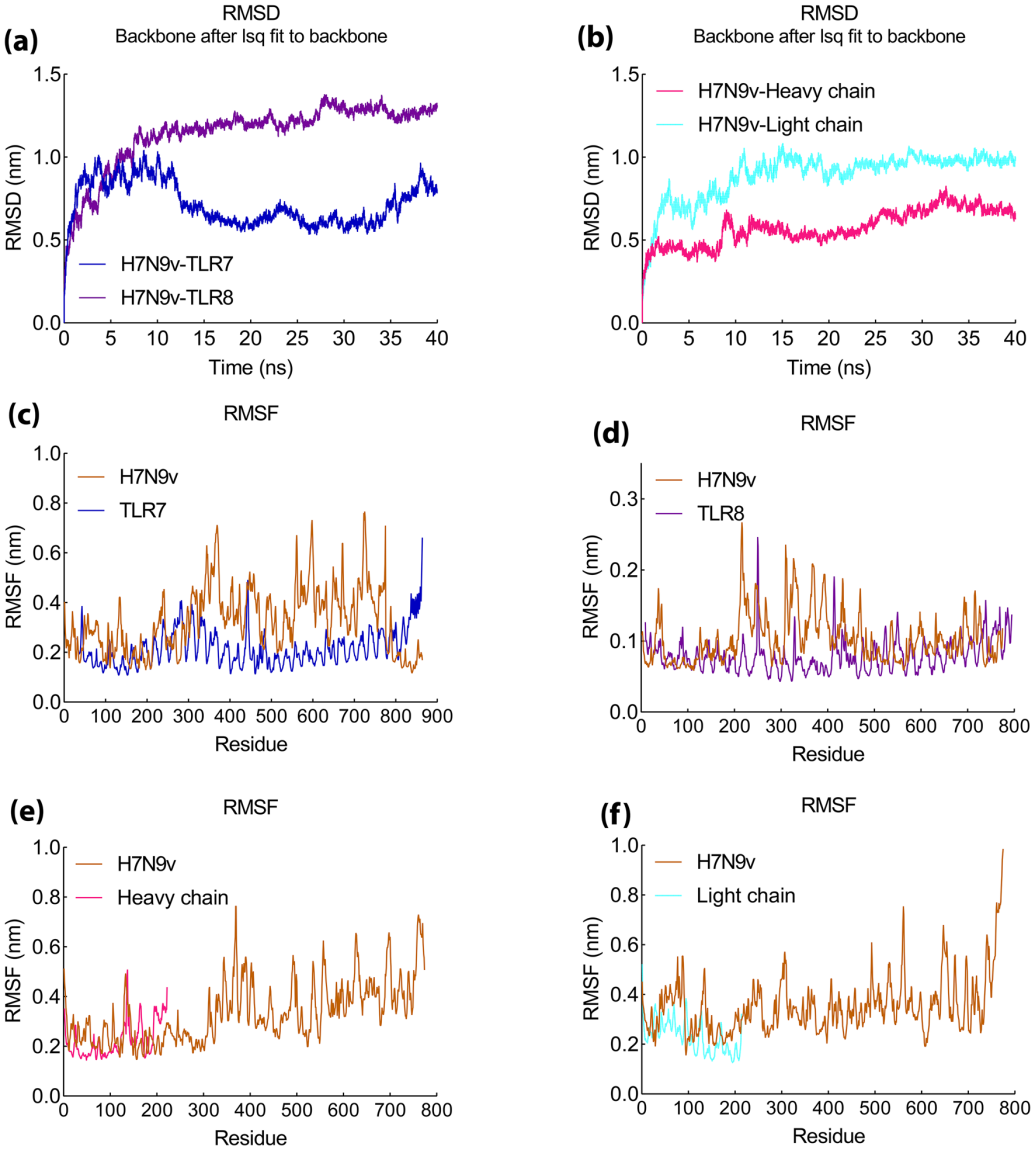
MD simulation results of H7N9v with proteins complex. (**a**) RMSD graph of H7N9vac-TLR7and H7N9vac-TLR8 complexes. (**b**) RMSD graph of H7N9vac-m826 Heavy chain H7N9vac-m826 Light chain complexes. (**c**), (**d**), (**e**) and (**f**) RMSF graph of H7N9vac-TLR7, H7N9vac-TLR8, H7N9vac-m826 Heavy chain and H7N9vac-m826 Light chain complexes.

The complexes were simulated by MD for 40 ns. The MD simulation results showed that the hydrogen bond in the H7N9vac-TLR7 and H7N9vac-TLR8 complexes was formed with the RMSD of 0.5 and 1 nm, respectively (Fig. 11a). Also, RMSD for H7N9vac-m826 Heavy chain and H7N9vac-m826 Light chain complexes were 0.6 and 0.9 nm, respectively (Fig. 11b).

The RMSF diagram indicated the residues in the H7N9vac-TLR7 and H7N9vac-TLR8 complexes in the MD simulation have very low volatility (Fig. 11c,d). H7N9vac-m826 Heavy chain and H7N9vac-m826 Light chain complexes showed a RMSF about 0.4 to 0.5 nm (Fig. 11e,f). These results indicate the maintenance of H7N9 structure in binding with the TLR7, TLR8 and m826 Heavy/Light chain. The H7N9vac-TLR7 and H7N9vac-TLR8 complexes returned to equilibrium after 12 ns, while, H7N9vac-m826 Heavy chain and H7N9vac-m826 Light chain complexes showed equilibrium about 10 ns since MD starting.

The gyration radius related to the H7N9vac-TLR7, H7N9vac-TLR8, H7N9vac-m826 Heavy chain and H7N9vac-m826 Light chain complexes are shown in supplementary Figure S10 as well as the H-Bond overall results.

The binding affinity of H7N9vac-TLR7, H7N9vac-TLR8, H7N9vac-m826 Heavy chain and H7N9vac-m826 Light chain complexes was confirmed using MM/PBSA calculations. The energy of the bonds formed between the receptor and the vaccine in each complex is calculated and as shown in Table 6. The total binding energy in H7N9vac-TLR7 and H7N9vac-TLR8 complexes is − 793.544 ± 35 kJ/mol and 1574.578 ± 72 kJ/mol, respectively, which indicated a high affinity between H7N9vac and TLR7. However, the amount of positive energy in H7N9vac-TLR8 complex probably stems from the existence of salt bridges between the H7N9vac and TLR8 structures.

**Table 6:** MM/PBSA analysis of MD simulation results of H7N9vac-TLR7, H7N9vac-TLR8, H7N9vac-m826 Heavy chain and H7N9vac-m826 Light chain complexes.

MM/PBSA-complexes	H7N9vac-TLR7 kJ/mol	H7N9vac-TLR8 kJ/mol	H7N9vac-m826 Heavy chain kJ/mol	H7N9vac-m826 Light chain kJ/mol
van der Waal energy	− 285.137 ± 24	− 178.801 ± 18	− 300.517 ± 14	− 163.482 ± 25
Electrostatic energy	− 986.686 ± 83	987.490 ± 67	− 570.977 ± 89	− 1685.757 ± 160
Polar solvation energy	511.138 ± 94	793.597 ± 76	405.651 ± 65	1225.086 ± 139
SASA energy	− 32.860 ± 4	− 27.708 ± 2	− 37.311 ± 1	− 33.226 ± 2
Binding energy	− 793.544 ± 35	1574.578 ± 72	− 503.155 ± 67	− 657.380 ± 66

The H7N9vac-m826 Heavy chain and H7N9vac-m826 Light chain complexes also have binding energies of − 503.155 ± 67 kJ/mol and − 657.380 ± 66 kJ/mol, respectively, which indicates that the structure of the H7N9vac binds to both the heavy and light chains of m826 antibody.

Besides, further studies show that in addition to total binding energy, van der Waals and electrostatic energies have also influenced the complexes that the contribution of van der Waals energy in the H7N9vac-TLR7 and H7N9vac-TLR8 complexes is − 285.137 ± 24 kJ/mol and 178.801 ± 18 kJ/mol, respectively. The electrostatic energy was − 986.686 ± 83 kJ/mol for H7N9vac-TLR7 complexes as well as 987.490 ± 67 kJ/mol for H7N9vac-TLR8.

van der Waal energy and Electrostatic energy for the H7N9vac-m826 Heavy chain complex were − 300.517 ± 14 and − 570.977 ± 89, respectively. In addition, the H7N9vac-m826 Light chain complex had van der Waal energy and Electrostatic energy equal to − 163.482 ± 25 and − 1685.757 ± 160, respectively. Generally, these results indicate that both complexes are stable, especially in H7N9vac-TLR7, H7N9vac-m826 Heavy chain and H7N9vac-m826 Light chain complexes.

### Immune simulation

**Figure 12:**
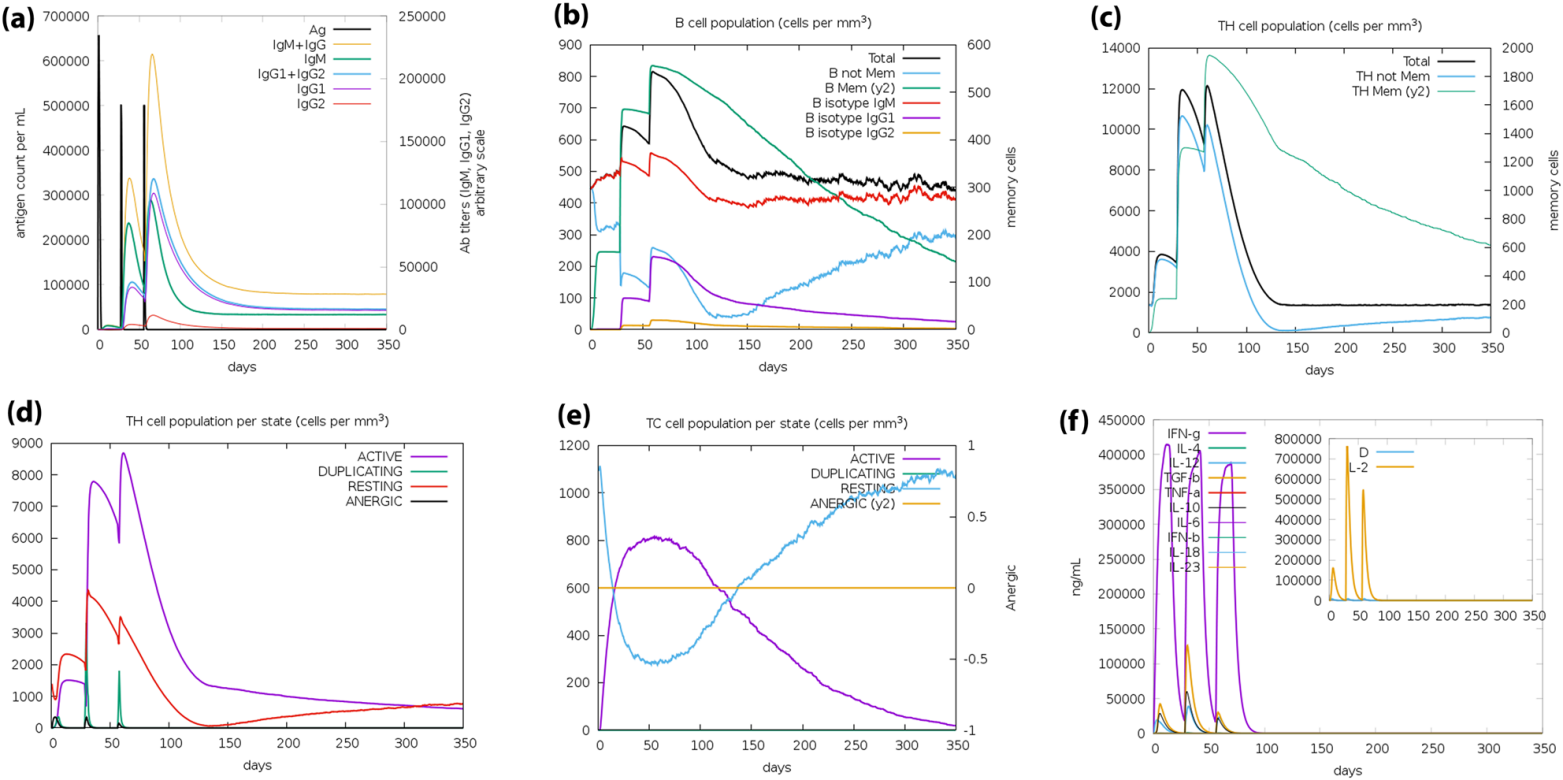
Simulation of the immune response against H7N9vac construct. (**a**) Immunoglobulins level after incitement by H7N9v construct. (**b**) Amount of B memory (y2) and total B isotype (IgM, IgG1, IgG2) as the immune response. (**c**) CD4 T-helper lymphocytes as a function of T-helper evolution. (**d**) Showing total and memory T-helper lymphocytes. (**e**) CD8 T-cytotoxic lymphocytes cell populations per state in response to antigen injection. (**f**) Produced IFN-γ concentration and IL-4, IL6 and IL12.

The results of online server C-ImmSim showed increased production of secondary immune responses consistent with the actual immune response. Increased levels of IgG2 antibodies and total IgM + IgG1 also increased as the immune response increased (Fig. 12a). The amount of B memory (y^2^) also increased with decreasing IgG1 and IgG2 isotypes (Fig. 12b). Besides, it has been shown that as TH memory (y^2^) increases (Fig. 12c), TH and TC cell populations increase (Fig. 12d). Finally, the produced IFN-γ concentration and TH cell population were increased (Fig. 12e,f). The results show that the simulated immune response immunization by the Influenza H7N9vac on the C-ImmSim server corresponds to the actual immune response.

## Discussion

All of the influenza A virus subtypes that have been identified so far belong to the waterfowl. This indicates that birds can be a natural host of the influenza A virus^29^. Studies have shown that the H7N9 virus is derived by reassortment between antigens expressed on the surface of the virus (H7N9) from wild birds and antigens expressed on the internal of the virus (H9N2) in poultry^6^. So far, 5 waves of epidemics have been caused by H7N9^30,31^. Reports indicate that the first wave of epidemic viruses is most similar to the first virus discovered from this lineage (A/Shanghai/1/2013)^32,33^.

Phylogenetic tree analysis for both HA and NA antigens showed that all H7N9 viruses in this study were located in polytomous clades with a common ancestor. HA and NA are spread in three continents of America, Europe and Asia. However, A/Shanghai/2/2013 influenza H7N9 from China, as human-derived influenza H7N9 along with avian and environmental influenza H7N9, associated with a common ancestor in the phylogenetic tree. Further studies of source diversity show that more than 81% of HA and NA influenza H7N9 is avian-mediated. Human-mediated H7N9 influenza is also approximately 15% and 16% related to HA and NA, respectively. Influenza virus transmission from birds to humans is rare due to host range restrictions. Besides, studies have shown that influenza A viruses select birds' intestinal tract for replication, while in humans, the virus replicates in the respiratory system. But reports confirm the transmission of H7N9, which can cause infection in humans. Although sustained transmission of avian influenza from human to human has not been reported, the occurrence of mutations in which the virus can adapt to human circulating, along with the lack of protective antibodies for humans, raises the risk of an H7N9 virus pandemic^34,35^. Furthermore, immune selection pressures, along with natural selection pressures, are the most important causes of antigenic drift and antigenic shift in IAVs, causing them to evolve continuously. As a result of this continuous evolution, the influenza epidemic and recurring pandemics occur, which can have very serious consequences for human society, such as the Covid-19 pandemic^36^.

Here, is introduced a multi-epitope vaccine against the H7N9 virus including immunogenic peptides of HA and NA proteins. HA protein, which is the most important determinant of antigen and viral entry into the host cell. NA protein was also used in the design of the vaccine, which is known for contributing to the release of the influenza virus into cell^37^. We utilized immunoinformatics tools to develop multi-epitope vaccines that are capable of producing humoral and cellular immunity. The multi-epitope vaccine provides its immunogenicity advantage, based on short immunogenic sequences containing appropriate immune responses. This method can prevent antigen overload as well as allergic responses in the host^38^. Total antigen sequence analysis can be performed using immunoinformatics and molecular instruments to investigate the possibility of T cell receptor (TCR) binding to host immune proteins^39^.

One of the unique features of multi-epitope vaccines over traditional vaccines is that multi-epitope vaccines can induce both humoral and cellular immune responses due to the presence of both B cell and T cell epitopes^40^. For screening of predicted CTL and HTL epitopes, special immunological characteristics such as antigenicity, immunogenicity and ability to bind to several MHC class I and MHC class II alleles were considered. T cell epitopes were predicted and it was found that a vaccine candidate made using the appropriate epitope could provide adequate immunogenicity if expressed.

B-cell epitopes are regions on the surface of antigens that specific antibodies recognize and attach to and stimulate the immune response^41–43^. The ability to identify these binding sites in the antigen sequence or structure is important for the development of recombinant vaccines^44^. Linear epitopes, which consist of a linear sequence of residues and conformational epitopes, which are composed of residues that are not interconnected in the native protein sequence, but are assembled by the folded protein structure^43^. However, the vast majority of B cell epitopes are conformational estimated and to a lesser extent linear^45^. Using BepiPred-2.0 and iBCE-EL servers, we identified linear B cell epitopes and included them in the final vaccine construct. We also used HA and NA crystallographic structures to identify the conformational epitopes obtained from them and after determining the overlap with the linear B cell epitopes, we included them in the final structure to predict the maximum responses related to humoral immunity.

HA, as a well-known trimeric surface glycoprotein, is the primary target for the development of antiviral vaccines against influenza virus. HA can bind to sialic acid receptors through the globular head region of HA1 subunit, thereby facilitating the entry of the virus into the cell^46,47^. In addition, HA causes fusion between viral and cell membranes under the influence of structural changes caused by acidic pHs. It is noteworthy that most influenza antibodies produced by immunization or infection are directed against five antigen sites on the HA1 globular head, Ca1, Ca2, Cb, Sa, and Sb^48–50^. In addition, studies have shown that mAbs are able to target the stem region of HA2 and exert their antiviral effect through Fc-Fcγ receptor interactions and antibody-dependent cell-mediated cytotoxicity (ADCC)^46,51–54^.

Analysis of the immunogenic properties of a HA1-specific fully human monoclonal antibodies (hmAbs) called m826 has shown that m826 binds specifically to hemagglutinin H7 (HA1). The m826 has minimal divergence from their germline predecessors, selected from a big naive antibody library created from the blood of healthy adult donors, and specifically targeting H7N9^46^. Interestingly, Yu et al. Showed that the m826 antibody induces very strong antibody-dependent cytotoxicity (ADCC) activity and is highly effective against H7N9 virus infection in the mouse model, and is completely protects mice exposed to the lethal challenge H7N9 virus via mechanisms that may include ADCC^46^. They also used crystal structure to show that m826 binds with a pH-dependent high affinity to a unique epitope distinct from the common HA1 antigen sites identified in the H7N9 HA1 and m826 complexes^46^. m826 is a germline antibody. this unique feature demonstrates that m826 as a promising therapeutic candidate, provides a tool to study the mechanisms of inhibition of virus infection by antibodies that could be a model for H7N9 vaccine immunogens as well as have the potential to be developed as antiviral agents for vaccine candidates^46^. In our immunoinformatics studies, we included HA1 B-cells epitopes involved in the heavy and light chain of m826 antibody in our construct, based on the crystallographic structure of m826 in complex with H7N9 HA1.

Prolonged immune responses can be enhanced by adding adjuvants to multi-epitope vaccines^55^. The multi-epitope vaccines can keep us away from the dangers of working directly with antigens or pathogens factors in vitro^56,57^. The multi-epitope vaccines, in addition to proving their efficacy in vivo by inducing protective immunity, have also entered phase I clinical trials^58,59^.

The EAAAK linker was used to link the adjuvants to each other and attach them to the construct. As rigid linkers between protein domains, EAAAK provides an Alpha helix-forming structure that increases stability, maintains a constant distance, and maintains the independent function of domains^60^. MHC-I epitopes were linked through AAY. The AAY linker helps to form epitopes in a natural form and does not allow the formation of junctional epitopes, which improves the presentation of the epitope^61,62^. CTL and HTL epitopes were fused via HEYGAEALERAG to bring an enhanced epitope presentation for the vaccine. HEYGAEALERAG can provide this property through the creation of a specific cleavage target for Proteasomal and lysosomal degradation systems^63,64^. The GPGPG linker was used to link MHCII epitopes. GPGPG is a glycine-rich linker that in addition to increasing construct solubility, provides freely of activity and high accessibility and flexibility for adjacent domains^65^. B-cell epitopes were bonded to each other via KK linkers. The KK linker of the lysosomal protease target sequence is cathepsin B, which is one of the important proteases for antigen processing in the field of MHC-II antigen presentation^66,67^. Linked peptides by KK can help to specially presentation of each peptide to antibodies via avoiding of inducing antibodies into the amino acid sequence that results from the binding of the two peptides^67^. Finally, the RVRR linker was embedded in the C-terminal construct, where it provides the binding of the vaccine to the His-tag sequence^68^.

The allergenicity and toxicity of the construct designed using the H7N9vac were evaluated using the AllerTOP v.2.0 and ToxDL servers, respectively, and it was confirmed that this protein is non-allergenic and non-toxic. The ProtParam tool provided by the ExPASy server was used to analyze the physicochemical properties of the designed construct. The M/W of the structure was ~ 86.38 kDa and pI was equal to 9.80. The aliphatic index indicated the thermal resilience of the construct after expression. The estimated instability index puts the H7N9vac’s protein in the classification of stable proteins. Because a protein with an instability index of less than 40 is considered stable, and if it tends to be more than 40, it is predicted to be unstable. The computed GRAVY index of the H7N9vac was considered as a reflection of the polar nature of H7N9vac that its effective interaction with water a lower score indicates the high solubility of this structure.

Structural validation of H7N9vac via Z-score by web server ProSA score (− 9.29), indicated that this protein can be expected to fall into the category of proteins that include Z-scores of structures determined by X-ray crystallographic experiments (more than 200 amino acids). These results confirmed the overall quality of H7N9vac. Evaluation of the third structure using Ramachandran diagram analysis showed that the maximum amino acids (99.1% residual) were in the allowed range, so the third validity ofH7N9vac structure is proven.

Adding adjuvants to a subunit vaccine can help increase the immune response it produces. Adjuvants can act as agonists for TLRs and can act as an effector to expand antibody recognition^69^. It has been indicated IVA can be recognized by TLR7 and TLR8 because of the TLR7 and TLR8 ability in coupling to ssRNAs^70^. TLR-7 has been shown to induce a humoral cellular response and long-term memory response in response to the ssRNA-mediate (Influenza and HIV) infection and vaccination^71,72^. Besides, TLR7 and TLR8 synthetic agonists have been shown to potentiate innate and adaptive immune responses^73^.

Recognition of ssRNA by TLR7 is primarily associated with the activation of downstream pathways of myD88, which can lead to IFN-α antiviral-cytokine and IL23/IL-1β responses (related to LT CD4^+^ T_H17_ and T-helper17) via IRF7 and mitogen-activated protein kinase (MAPK), respectively. Also TLR 8, after recognition and binding to ssRNA, is associated with the activation of downstream pathways of myD88, which activates IRF3, IFN-B responses, and stimulates B cell-mediated humoral immunity. B cell epitopes are recognized by B cell receptors or secretory antibodies and play a very important role in creating an efficient immune response. Both TLR7 and TLR 8, which are referred to as fraternal twins, are jointly involved in myD88-dependent NF-kB activation, ultimately producing IL-12 and stimulating LT CD4^+^ T_H1_ and T-helper1 cellular immunity^74–79^.

In this regard, HβD-3 was used as an adjuvant, to take advantage of its synergistic and interactive properties with other TLRs and pathways associated with Myd88 downstream, as well as activating the expression of stimulatory molecules in antigen-presenting cells in this pathway^80,81^. Besides, studies have shown that HβD-3, through TLR1/TLR2-mediated pathways, can induce IFN-γ secretion associated with the cytokine Th-1; in contrast, it is independently involved in the secretion of interferon-gamma (IFN-γ) and the activation of natural killer (NK) cells^82,83^.

The interaction of H7N9vac protein structures with TLR7 and TLR8 structures was analyzed by protein–protein docking. Analysis of H7N9vac-TLR7 and H7N9vac-TLR8 complexes showed that 6 and 4 hydrogen bonds involved these interactions, respectively. Also, 4 and 6 hydrogen bonds were involved in the interaction of H7N9vac-m826 Heavy chain and H7N9vac-m826 Light chain complexes, respectively.

The H7N9vac-TLR7 and H7N9vac-TLR8 complexes showed that proteins interact with each other through the formation of hydrogen bonds. The H7N9vac-TLR7 and H7N9vac-TLR8 complexes RMSD diagrams showed that the simulation was quite stable. However, the mean RMSD of 0.6 nm for the H7N9vac-TLR7 complex is higher than the mean RMSD of 0.3 nm for the H7N9vac-TLR87 complex, indicating that the intermolecular energies in the H7N9vac-TLR8 complex are higher. H7N9vac-m826 Heavy chain and H7N9vac-m826 Light chain complexes showed they are close to each other and mimic each other in RMSD. Also, the graph of gyration shows the amino acid's consistency in their radial axis and they do not go beyond the range of their radius axis, which confirms the stability of the complex during the simulation. The RMSF diagram is also consistent with the results; implying that the amino acids of the H7N9vac-TLR7, H7N9vac-TLR8, H7N9vac-m826 Heavy chain and H7N9vac-m826 Light chain complexes binding sites maintain their stability during the simulation. In general, docking and MD results showed that the complexes can maintain their stability during the simulation. The obtained results in the present study showed that this recombinant construct can help stimulate TLR7/8. Binding of the heavy and light chain of m826 antibody to the residues of the vaccine structure showed that the production of antibodies against the proposed vaccine in this study is to be expected. Because it's important to have the correct 3D structure of the antigen to be recognized by the humoral immune system. Therefore, the B-cell epitopes in the 3D vaccine structure need to mimics the correct fold in the native structure of the antigen to induce the same immune response^46^.

Besides, the MM/PBSA results showed that in addition to the total energy, the complexes are also affected by the energies resulting from covalent and electrostatic bonds. However, the positive binding energy generated in H7N9vac-TLR8 complex can be affected by the salt bridges in the interaction between the vaccine structure and TLR8. Studies have shown that although salt bridges may help stabilize protein associations, they also destabilize protein cores^84^. In addition, the findings indicate that the salt bridge interactions between acidic amino acids (Glu^−^ and Asp^−^) and alkaline amino acids (Arg^+^, Lys^+^, and His^+^) are the strongest residual residue interactions. But, may be weakened by the effects of solvation and broken down by lower pH conditions^85^. These findings are consistent with our results. As seen in H7N9vac-TLR8, salt bridges are formed between glutamine-arginine, aspartate-lysine, and lysine-glutamine and may have affected interactions between vaccine and TLR8.

Influenza viruses are ssRNA that, in addition to the humoral immunity induced by B cells, are also required to activate T cell-dependent immune responses to inhibit intracellular survival and proliferation. As the results of the C-ImmSim Online server prediction show, the development of B cells and T cells caused long-term memory. IgG1 and IgG2, which are indices of Th1 and Th2 response to influenza antigens, respectively, are involved in the protection against influenza infection^86,87^. Previous studies have shown that both human and avian hosts show a decrease in T cell populations in response to AI infection^88^. Decreased T lymphocytes are often associated with the upregulation of proinflammatory cytokines such as interferons (IFNs), especially IFN-γ, as well as interleukins (ILs) such as IL-6, which eventually lead to hypercytokinemia and Activation of cell apoptosis^89^. Elevated levels of IL-12 and IFN-γ are due to strong cellular immunity. Although studies have shown that T epitopes of H7N9 strains mimicking human sequencing are associated with attenuated IFN responses in humans, the ICM server results in an excellent stimulation of FN-γ by this shows the vaccine^90–92^. However, ICM server results indicate that the increase in the number of Th1 cells is associated with an increase in the level of Cytotoxic T cells.

## Material and methods

### Protein sequences retrieval and phylogenetic evolution

The human-isolated H7N9 influenza virus was considered as a well-known H7N9 for this study^93^. Hemagglutinin and Neuraminidase from Influenza A virus (A/Shanghai/02/2013(H7N9)) were retrieved from NCBI with YP_009118475.1 and YP_009118481.1 reference sequences, respectively.

The HA and NA protein sequences of the A/Shanghai/02/2013 (H7N9) strain were compared and analyzed along with the HA and NA protein sequences of different H7N9 strains in the NCBI Influenza Virus Resource (https://www.ncbi.nlm.nih.gov/genomes/FLU/Database/nph-select.cgi?go=database). The phylogenetic tree was constructed so that 119 out of 761 sequences (16%) for HA protein and 121 out of 741 sequences (16%) for NA protein were excluded in total amino acid sequences based on the NCBI Influenza Virus Resource default parameters. Finally, the phylogenetic trees were drawn through the clustering algorithm of the Neighbor-Joining and F84 distance for protein. The phylogenetic trees were visualized and adjusted in iTOL v5 (https://itol.embl.de/) and Illustrator, respectively.

### Linear and conformational B Cell epitope prediction

B cell epitopes are one of the most important factors contributing to the vaccine design because they are characterized by the immune system. In this study, we used BepiPred-2.0 (http://www.cbs.dtu.dk/services/BepiPred/) to identify linear B Cell epitope on HA and NA protein sequences^43^. BepiPred-2.0 is a web server for predicting B-cell epitopes from antigen sequences. BepiPred-2.0 is based on a random forest algorithm trained on epitopes annotated from antibody-antigen protein structures. BepiPred-2.0 utilizes sequence-based epitope prediction both on epitope data derived from 3D structures, and on a large collection of linear epitopes downloaded from the IEDB database.

Also, iBCE-EL (http://thegleelab.org/iBCE-EL/) was used to confirm B-cell epitopes which were selected by BepiPred-2.0. iBCE-EL is a web based prediction server for linear B-cell epitope. It is an ensemble method that combined extremely randomized tree and gradient boosting algorithms, which respectively utilizes a combination of amino acid composition and physicochemical properties and a combination of dipeptide and physicochemical properties as an input features. For a given peptide, iBCE-EL predicts its calss and probability values^95^.

It is estimated that more than 90% of B cell epitopes are discontinuous; implying they contain sequences that are far from each other by long-distance pathogenic protein sequences but are adjacent by protein folding properties. From the server ElliPro (http://tools.iedb.org/ellipro/) B cell epitopes were used to predict the three-dimensional structure.

### Cytotoxic T cell epitope

Prediction of T cell epitopes was performed using an improved neural network method provided by the NetMHC—4.0 server at https://services.healthtech.dtu.dk/service.php?NetMHC-4.0. All epitopes were considered as 9mers-long for cytotoxic T Cell prediction (recommended from server), which are recognized by the HLA Class including HLA-B, HLA-B, and HLA-C molecules. The threshold for strong and weak binders was adjusted 0.5 and 2, respectively.

### Helper T cell epitope

The NetMHCII 2.3 server was used to predict the binding of epitopes MHC-II. The NetMHCII 2.3 server utilizes an artificial neuron network for predicting epitope affinity for HLA-DR, HLA-DQ, HLA-DP molecules.

### TAP transport/Proteasomal cleavage

NetCTL 1.2 Server was used to predict transporter associated with antigen processing (TAP) and Proteasomal cleavage. The server uses an algorithm that simultaneously provides an integrated prediction of the peptide MHC class I binding, TAP transport efficiency and Proteasomal C terminal cleavage. The default settings were considered as weight in C terminal cleavage equal 0.15 and Weight on TAP transport efficiency equal 0.05.

### IFN-γ cytokine inducer prediction

Prediction of IFN-gamma cytokine inducer epitopes was performed via the online server http://crdd.osdd.net/raghava/ifnepitope/index.php. IFN-γ is considered an important factor in inducing intracellular Th1 immune responses that elicit antiviral activity in both adaptive and innate immune systems. Using this server, MHC class II-binding peptides capable of inducing IFN-γ from CD4 + T cells were predicted and settings were adjusted so that Support Vector Machine (SVM) based was select on IFN-γ versus other cytokines.

### Determination of population coverage

Population coverage tool in IEDB server (http://tools.iedb.org/population) was utilized for determining the coverage rate of the population for any selected epitope. Population coverage estimation can help in an appropriate epitopes prediction for different HLA binding. Epitopes with increased HLA binding can coverage different frequencies in different ethnicities which leads to eliminating denominated MHC restriction of T cell responses. In this regard, MHC-I HLA binding including HLA-B5701, HLA-B5802, HLA-B5801, HLA-B2705, HLA-A2603, HLA-A0203, HLA-B8301, HLA-A3002, HLA-B4002, HLA-B4402, HLA-A2902, HLA-C0401 and HLA-C0303 as well as HLA-A0203, HLA-A6601, HLA-A2402 and HLA-B1517 were evaluated for HA and NA proteins, respectively. Besides, MHC-II HLA binding including DRB1_0101, DPA10103-DPB10301, DPA10201-DPB10501, DQA10501-DQB10201, DPA10103-DPB10401, DRB1_0403 as well as DRB1_0404, DRB1_0402, DRB1_0403, DQA10102-DQB10501, DRB1_1302, DRB1_0401, DQA10401-DQB10402 and DQA10301-DQB10302 were evaluated for HA and NA proteins, respectively.

### Epitopes conservation analysis

IEDB conservancy analysis tool was considered a monitoring tool for investigation of the degree of conservation in the selected epitopes in comparison to other similar sequences. Epitopes are showing with minimum 0 and maximum 100 percent conservation in different lengths.

### Insilico cloning and vaccine optimization

The codon optimization for recombinant construct expression in the pET-26b(+) vector was performed applying the Java Utility Server (JCat) (http://www.prodoric.de/JCat). Given that the expression host used in the present study is different from the native influenza host, the codon optimization was adjusted based on the codon preference of influenza structure in *E. coli* K12. The CAI score and GC content are ideally 1.0 and 30–70% respectively, which are used to predict protein expression measures. Using the SnapGene tool, the sites of the *Nde*I and *Xho*I restriction enzymes were identified on the optimized nucleotide sequence, and the recombinant influenza gene sequence was inserted into the pET-26b(+) vector.

EAAAK linkers were used to link HβD-3 and PADRE adjuvants at the N-terminal construct. Then AAY linkers bonded CTL epitopes to each other. The HEYGAEALERAG linker was placed as the interface between CTL epitopes and HTL epitopes. The GPGPG linkers also acted as the link between HTL epitopes. The KK linkers were used to stick B-cell epitopes. Finally, RVRR bonded construct to HisTag sequence in C-terminal of vaccine construct.

Prediction of disulfide bond formation in construct was performed via DiANNA 1.1 server (http://clavius.bc.edu/~clotelab/DiANNA/main.html). This server utilizes a SVM, along with an architecture neural network state-of-the-art method to determine cysteine species and disulfide connectivity.

Physicochemical, antigenicity, allergenicity, toxicity properties of the vaccine.

ProtParam tool in the ExPASy server (https://web.expasy.org/protparam/) provided a prediction of physicochemical properties for the final protein construct. VaxiJen v2.0 server on http://www.ddg-pharmfac.net/vaxijen/VaxiJen.html was used for predicting the antigen. The VaxiJen is considered an independent alignment method. Server accuracy varies from 70 to 89%^96^. Total protein sensitivity was calculated by AllerTOP v. 2.0 (https://www.ddg-pharmfac.net/AllerTOP/). AllerTOP v. 2.0 server benefits from an auto cross-covariance (ACC) and k-nearest neighbor algorithm (kNN, k = 1) to predict allergenicity of protein in terms of hydrophobicity, molecular weight, secondary structure properties and relative abundance of amino acids. ToxDL server (http://www.csbio.sjtu.edu.cn/bioinf/ToxDL/index.html) was utilized to determine protein toxicity. ToxDL server utilizes an interpretable deep learning-based method to classify proteins in two toxic and non-toxic based on multimodal method including three component of CNNs, InterProscan database and word2vec encoder protein domain.

### Modeling, refinement, and validation of vaccine construct

RoseTTAFold online software (https://robetta.bakerlab.org/) was used to build the 3D structure. RoseTTAFold is a “three-track” neural network, meaning it simultaneously considers patterns in protein sequences, how a protein’s amino acids interact with one another, and a protein’s possible 3D structure and achieved accuracies approaching those of DeepMind. In RoseTTAFold architecture, one-, two-, and three-dimensional information flows back and forth, allowing the network to collectively reason about the relationship between a protein’s chemical parts and its folded structure^97^.

To ensure the accuracy of the simulated building, http://services.mbi.ucla.edu/PROCHECK and http://services.mbi.ucla.edu/SAVES were used. The PROSA server (https://prosa.services.came.sbg.ac.at/prosa.pHp) determined the Z-Score point and protein energy balance. The Ramachandran plot graph was drawn by the online website http://molprobity.biochem.duke.edu/. Chimera V 1.13.1 software determined the best spatial resolution for the optimal energy used in the present study. The website of http://galaxy.seoklab.org/cgi-bin/submit.cgi?type=REFINE was used to structure refinement.

### The prediction of protein secondary structure

NetSurfP-2.0 online software (https://services.healthtech.dtu.dk/service.php?NetSurfP-2.0) was used for predicting the second structure. NetSurfP-2.0 as a sequence-based tool provides a prediction of amino acids secondary structure so that with utilizing a deep machine learning it combined predicted structural features of amino acids including disorders, Phi/Psi angles and surface accessibility to visualizes a protein secondary structure.

### Molecular docking of vaccine constructs with TLR7 and TLR8 receptor

TLR7 (PDB code: 7CYN), TLR8 (PDB code: 3w3g) and human germline monoclonal antibody (m826) (PDB code: 5vag) 3D structures were retrieved from RCSB server. Then, the 3D structures of TLRs and m826 were corrected in PyMOL v2.3.4 software for energy. All water molecules were removed from the PDB structure of the vaccine, TLRs and m826. After preparing the 3D structure in Chimera V 1.13.1 software, Influenza constructs, TLR7, TLR8 and m826 were submitted to the HADDOCK server so that the interaction regions will be identified. The highest rankings for each complex were selected at the lowest intermolecular binding of the whole-molecular influenza-TLRs and whole-molecular influenza- m826 heavy and light chains from the HADDOCK with the lowest mean RMSD. H-band formation was assessed by the LigPlot^+^.

### Molecular dynamics and MM/PBSA analysis

The molecular dynamics calculation of the vaccine-TLR7/8 and vaccine-m826 complexes were performed by Gromacs v 4.6.5 and CHARMM36 all-atom force field. Protein–protein complexes were filled with water molecules and then the system was neutralized. The energy minimization of the system was done. The NVT and NPT optimization were done at a temperature of 300 K, the pressure of 1 bar and restraint forces of 1000 kJ/mol. Then, molecular dynamics simulation was performed for 40,000 ps. All bonds were constrained by applying the LINCS algorithm during the simulation.

### Immune simulations of vaccine construct

Simulating and predicting the immune response of recombinant influenza structure was performed using the C-ImmSim server. C-ImmSim used an agent-based model for machine learning techniques and immune epitope prediction that utilized a position-specific scoring matrix (PSSM) that allowed it for the prediction of immune interactions. C-ImmSim simulates three distinct anatomical regions for mammals simultaneously. These include bone marrow (to simulate hematopoietic stem cells, lymphoid cells and myeloid cells), thymus (native T cell selection), and the third lymph node, such as the lymph node. Taking into account the 4-week interval between the first and second dose of the vaccine, the Simulation Steps parameter was set to 1050. Then 3 injections were considered so that time-steps were set at 1, 84 and 168, while an one time-step is equal to eight hours of real life. Other parameters were applied by default in this study.

## Supplementary Information

Supplementary Information.

## References

[1] Chen Y (2013). Human infections with the emerging avian influenza A H7N9 virus from wet market poultry: Clinical analysis and characterisation of viral genome. Lancet (London, England).

[2] Dortmans JC (2013). Adaptation of novel H7N9 influenza A virus to human receptors. Sci. Rep..

[3] Su S (2017). Epidemiology, evolution, and pathogenesis of H7N9 influenza viruses in five epidemic waves since 2013 in China. Trends Microbiol..

[4] Vasin AV (2014). Molecular mechanisms enhancing the proteome of influenza A viruses: An overview of recently discovered proteins. Virus Res..

[5] Petrova VN, Russell CA (2018). The evolution of seasonal influenza viruses. Nat. Rev. Microbiol..

[6] Liu D (2013). Origin and diversity of novel avian influenza A H7N9 viruses causing human infection: Phylogenetic, structural, and coalescent analyses. The Lancet.

[7] Su S (2015). Epidemiology, evolution, and recent outbreaks of avian influenza virus in China. J. Virol..

[8] Zhang J (2020). Evolution and antigenic drift of influenza A (H7N9) viruses, China, 2017–2019. Emerg. Infect. Dis..

[9] Su S (2016). Epidemiology, genetic recombination, and pathogenesis of coronaviruses. Trends Microbiol..

[10] Nachbagauer R (2021). A chimeric hemagglutinin-based universal influenza virus vaccine approach induces broad and long-lasting immunity in a randomized, placebo-controlled phase I trial. Nat. Med..

[11] Du L (2013). A critical HA1 neutralizing domain of H5N1 influenza in an optimal conformation induces strong cross-protection. PLoS ONE.

[12] Aguilar-Yáñez JM (2010). An influenza A/H1N1/2009 hemagglutinin vaccine produced in *Escherichia coli*. PLoS ONE.

[13] To KK (2015). Recombinant influenza A virus hemagglutinin HA2 subunit protects mice against influenza A(H7N9) virus infection. Arch. Virol..

[14] Fan X (2015). Targeting the HA2 subunit of influenza A virus hemagglutinin via CD40L provides universal protection against diverse subtypes. Mucosal Immunol..

[15] Yang H, Carney PJ, Chang JC, Villanueva JM, Stevens J (2013). Structural analysis of the hemagglutinin from the recent 2013 H7N9 influenza virus. J. Virol..

[16] Wagner R, Matrosovich M, Klenk H-D (2002). Functional balance between haemagglutinin and neuraminidase in influenza virus infections. Rev. Med. Virol..

[17] Bangaru S (2019). A site of vulnerability on the influenza virus hemagglutinin head domain trimer interface. Cell.

[18] Xu Y (2019). Avian-to-human receptor-binding adaptation of avian H7N9 influenza virus hemagglutinin. Cell Rep..

[19] de Vries RP (2017). Three mutations switch H7N9 influenza to human-type receptor specificity. PLoS Pathog..

[20] Xu Q, Chen Z, Cheng X, Xu L, Jin H (2013). Evaluation of live attenuated H7N3 and H7N7 vaccine viruses for their receptor binding preferences, immunogenicity in ferrets and cross reactivity to the novel H7N9 virus. PLoS ONE.

[21] Gao R (2013). Human infection with a novel avian-origin influenza A (H7N9) virus. N. Engl. J. Med..

[22] Rudenko L (2016). H7N9 live attenuated influenza vaccine in healthy adults: A randomised, double-blind, placebo-controlled, phase 1 trial. Lancet Infect Dis..

[23] Stadlbauer D (2021). AS03-adjuvanted H7N9 inactivated split virion vaccines induce cross-reactive and protective responses in ferrets. npj Vaccines.

[24] Oshansky CM (2021). Adjuvanted recombinant hemagglutinin H7 vaccine to highly pathogenic influenza A(H7N9) elicits high and sustained antibody responses in healthy adults. npj Vaccines.

[25] Gilchuk IM (2019). Influenza H7N9 virus neuraminidase-specific human monoclonal antibodies inhibit viral egress and protect from lethal influenza infection in mice. Cell Host Microbe.

[26] Solanki V, Tiwari M, Tiwari V (2019). Prioritization of potential vaccine targets using comparative proteomics and designing of the chimeric multi-epitope vaccine against *Pseudomonas aeruginosa*. Sci. Rep..

[27] Shey RA (2019). In-silico design of a multi-epitope vaccine candidate against onchocerciasis and related filarial diseases. Sci. Rep..

[28] Corder BN, Bullard BL, Poland GA, Weaver EA (2020). A decade in review: A systematic review of universal influenza vaccines in clinical trials during the 2010 decade. Viruses.

[29] Nelson MI, Vincent AL (2015). Reverse zoonosis of influenza to swine: New perspectives on the human–animal interface. Trends Microbiol..

[30] Watanabe T, Watanabe S, Maher EA, Neumann G, Kawaoka Y (2014). Pandemic potential of avian influenza A (H7N9) viruses. Trends Microbiol..

[31] Xu J, Lu S, Wang H, Chen C (2013). Reducing exposure to avian influenza H7N9. The Lancet.

[32] Wu A (2013). Sequential reassortments underlie diverse influenza H7N9 genotypes in China. Cell Host Microbe.

[33] Lu S (2014). Prognosis of 18 H7N9 avian influenza patients in Shanghai. PLoS ONE.

[34] Tong S (2013). New world bats harbor diverse influenza A viruses. PLoS Pathog..

[35] Murphy BR (1984). Avian-human reassortant influenza A viruses derived by mating avian and human influenza A viruses. J. Infect. Dis..

[36] Zhao R (2011). Identification of a highly conserved H1 subtype-specific epitope with diagnostic potential in the hemagglutinin protein of influenza A virus. PLoS ONE.

[37] Bui CM, Gardner L, MacIntyre R, Sarkar S (2017). Influenza A H5N1 and H7N9 in China: A spatial risk analysis. PLoS ONE.

[38] Sette A, Fikes J (2003). Epitope-based vaccines: An update on epitope identification, vaccine design and delivery. Curr. Opin. Immunol..

[39] Usman Mirza M (2016). Towards peptide vaccines against Zika virus: Immunoinformatics combined with molecular dynamics simulations to predict antigenic epitopes of Zika viral proteins. Sci. Rep..

[40] Moise L (2018). T cell epitope engineering: An avian H7N9 influenza vaccine strategy for pandemic preparedness and response. Hum. Vac. Immunother..

[41] Galanis KA (2021). Linear B-cell epitope prediction for in silico vaccine design: A performance review of methods available via command-line interface. Int. J. Mol. Sci..

[42] Wang X (2018). Evaluation and comparison of newly built linear B-Cell epitope prediction software from a users' perspective. Curr. Bioinform..

[43] Jespersen MC, Mahajan S, Peters B, Nielsen M, Marcatili P (2019). Antibody specific B-cell epitope predictions: leveraging information from antibody-antigen protein complexes. Front. Immunol..

[44] Russi RC, Bourdin E, García MI, Veaute CMI (2018). In silico prediction of T- and B-cell epitopes in PmpD: First step towards to the design of a *Chlamydia trachomatis* vaccine. Biomed. J..

[45] Kringelum JV, Nielsen M, Padkjær SB, Lund O (2013). Structural analysis of B-cell epitopes in antibody:protein complexes. Mol. Immunol..

[46] Yu F (2017). A Potent Germline-like human monoclonal antibody targets a pH-sensitive epitope on H7N9 influenza hemagglutinin. Cell Host Microbe.

[47] Huang K-YA (2019). Structure–function analysis of neutralizing antibodies to H7N9 influenza from naturally infected humans. Nat. Microbiol..

[48] Gerhard W, Yewdell J, Frankel ME, Webster R (1981). Antigenic structure of influenza virus haemagglutinin defined by hybridoma antibodies. Nature.

[49] Caton AJ, Brownlee GG, Yewdell JW, Gerhard W (1982). The antigenic structure of the influenza virus A/PR/8/34 hemagglutinin (H1 subtype). Cell.

[50] Zuo T (2015). Comprehensive analysis of antibody recognition in convalescent humans from highly pathogenic avian influenza H5N1 infection. Nat. Commun..

[51] Kallewaard NL (2016). Structure and function analysis of an antibody recognizing all influenza A subtypes. Cell.

[52] DiLillo DJ, Tan GS, Palese P, Ravetch JV (2014). Broadly neutralizing hemagglutinin stalk-specific antibodies require FcγR interactions for protection against influenza virus in vivo. Nat. Med..

[53] Henry Dunand CJ (2016). Both neutralizing and non-neutralizing human H7N9 influenza vaccine-induced monoclonal antibodies confer protection. Cell Host Microbe.

[54] Tan GS (2016). Broadly-reactive neutralizing and non-neutralizing antibodies directed against the H7 influenza virus hemagglutinin reveal divergent mechanisms of protection. PLoS Pathog..

[55] Zheng D (2016). Influenza H7N9 LAH-HBc virus-like particle vaccine with adjuvant protects mice against homologous and heterologous influenza viruses. Vaccine.

[56] De Groot AS, Moise L, McMurry JA, Martin W (2008). Epitope-based immunome-derived vaccines: A strategy for improved design and safety. Clin. Appl. Immunomics.

[57] Davies MN, Flower DR (2007). Harnessing bioinformatics to discover new vaccines. Drug Discovery Today.

[58] Toledo H (2001). A phase I clinical trial of a multi-epitope polypeptide TAB9 combined with montanide ISA 720 adjuvant in non-HIV-1 infected human volunteers. Vaccine.

[59] Zhou WY (2009). Therapeutic efficacy of a multi-epitope vaccine against *Helicobacter pylori* infection in BALB/c mice model. Vaccine.

[60] Chen X, Zaro JL, Shen W-C (2013). Fusion protein linkers: Property, design and functionality. Adv. Drug Deliv. Rev..

[61] Yang Y (2015). In silico design of a DNA-based HIV-1 multi-epitope vaccine for Chinese populations. Hum. Vac. Immunother..

[62] Reddy Chichili VP, Kumar V, Sivaraman J (2013). Linkers in the structural biology of protein–protein interactions. Protein Sci..

[63] Athanasiou E (2017). A poly(Lactic-co-Glycolic) acid nanovaccine based on chimeric peptides from different leishmania infantum proteins induces dendritic cells maturation and promotes peptide-specific IFNγ-producing CD8+ t cells essential for the protection against experiment. Front. Immunol..

[64] Nezafat N, Ghasemi Y, Javadi G, Khoshnoud MJ, Omidinia E (2014). A novel multi-epitope peptide vaccine against cancer: An in silico approach. J. Theor. Biol..

[65] Dong R, Chu Z, Yu F, Zha Y (2020). Contriving multi-epitope subunit of vaccine for COVID-19: Immunoinformatics approaches. Front. Immunol..

[66] Lennon-Duménil AM, Bakker AH, Wolf-Bryant P, Ploegh HL, Lagaudrière-Gesbert C (2002). A closer look at proteolysis and MHC-class-II-restricted antigen presentation. Curr. Opin. Immunol..

[67] Yano A (2005). An ingenious design for peptide vaccines. Vaccine.

[68] Safavi A, Kefayat A, Mahdevar E, Abiri A, Ghahremani F (2020). Exploring the out of sight antigens of SARS-CoV-2 to design a candidate multi-epitope vaccine by utilizing immunoinformatics approaches. Vaccine.

[69] Vogel FR (2000). Improving vaccine performance with adjuvants. Clin. Infect. Dis..

[70] Pulendran, B. & Maddur, M. S. in *Influenza Pathogenesis and Control - Volume II* (eds Michael B. A. Oldstone & Richard W. Compans) 23–71 (Springer, 2015).

[71] Jeisy-Scott V (2012). TLR7 recognition is dispensable for influenza virus A infection but important for the induction of hemagglutinin-specific antibodies in response to the 2009 pandemic split vaccine in mice. J. Virol..

[72] Wille-Reece U, Wu CY, Flynn BJ, Kedl RM, Seder RA (2005). Immunization with HIV-1 Gag protein conjugated to a TLR7/8 agonist results in the generation of HIV-1 Gag-specific Th1 and CD8+ T cell responses. J. Immunol. (Baltimore, Md.: 1950).

[73] Miller SM (2018). Investigation of novel TLR7/8 ligands in combination with TLR4 ligands as adjuvants to drive cell mediated anti-influenza immunity. J. Immunol..

[74] Georg P, Sander LE (2019). Innate sensors that regulate vaccine responses. Curr. Opin. Immunol..

[75] Zhang Z (2018). Structural analyses of toll-like receptor 7 reveal detailed RNA sequence specificity and recognition mechanism of agonistic ligands. Cell Rep..

[76] Tanji H (2015). Toll-like receptor 8 senses degradation products of single-stranded RNA. Nat. Struct. Mol. Biol..

[77] Heil F (2004). Species-specific recognition of single-stranded RNA via toll-like receptor 7 and 8. Science (New York, N.Y.).

[78] Ugolini M (2018). Recognition of microbial viability via TLR8 drives TFH cell differentiation and vaccine responses. Nat. Immunol..

[79] de Marcken M, Dhaliwal K (2019). TLR7 and TLR8 activate distinct pathways in monocytes during RNA virus infection. Sci. Signal..

[80] Harder J, Bartels J, Christophers E, Schröder J-M (2001). Isolation and characterization of human µ-Defensin-3, a novel human inducible peptide antibiotic. J. Biol. Chem..

[81] Funderburg N (2007). Human β-defensin-3 activates professional antigen-presenting cells via Toll-like receptors 1 and 2. Proc. Natl. Acad. Sci..

[82] Judge CJ (2015). HBD-3 induces NK cell activation, IFN-γ secretion and mDC dependent cytolytic function. Cell. Immunol..

[83] Netea MG, Van der Meer JWM, Sutmuller RP, Adema GJ, Kullberg B-J (2005). From the Th1/Th2 paradigm towards a toll-like receptor/T-helper bias. Antimicrobial Agents Chemother..

[84] Xu D, Tsai CJ, Nussinov R (1997). Hydrogen bonds and salt bridges across protein-protein interfaces. Protein Eng..

[85] Xie N-Z, Du Q-S, Li J-X, Huang R-B (2015). Exploring strong interactions in proteins with quantum chemistry and examples of their applications in drug design. PLoS ONE.

[86] Chen WH (2011). Antibody and Th1-type cell-mediated immune responses in elderly and young adults immunized with the standard or a high dose influenza vaccine. Vaccine.

[87] Palladino G, Scherle PA, Gerhard W (1991). Activity of CD4+ T-cell clones of type 1 and type 2 in generation of influenza virus-specific cytotoxic responses in vitro. J. Virol..

[88] Wang Z (2015). Recovery from severe H7N9 disease is associated with diverse response mechanisms dominated by CD8+ T cells. Nat. Commun..

[89] Wang Z (2014). Early hypercytokinemia is associated with interferon-induced transmembrane protein-3 dysfunction and predictive of fatal H7N9 infection. Proc. Natl. Acad. Sci. USA.

[90] To KK (2016). Human H7N9 virus induces a more pronounced pro-inflammatory cytokine but an attenuated interferon response in human bronchial epithelial cells when compared with an epidemiologically-linked chicken H7N9 virus. Virol. J..

[91] Liu R (2015). H7N9 T-cell epitopes that mimic human sequences are less immunogenic and may induce Treg-mediated tolerance. Hum. Vac. Immunother..

[92] Arilahti V, Mäkelä SM, Tynell J, Julkunen I, Österlund P (2014). Novel avian influenza A (H7N9) virus induces impaired interferon responses in human dendritic cells. PLoS ONE.

[93] Zhu H (2013). Infectivity, transmission, and pathology of human-isolated H7N9 influenza virus in ferrets and pigs. Science (New York, N.Y.).

[94] Jespersen MC, Peters B, Nielsen M, Marcatili P (2017). BepiPred-2.0: Improving sequence-based B-cell epitope prediction using conformational epitopes. Nucl. Acids Res..

[95] Manavalan B, Govindaraj RG, Shin TH, Kim MO, Lee G (2018). iBCE-EL: A new ensemble learning framework for improved linear B-cell epitope prediction. Front. Immunol..

[96] Doytchinova IA, Flower DR (2007). VaxiJen: A server for prediction of protective antigens, tumour antigens and subunit vaccines. BMC Bioinform..

[97] Baek M (2021). Accurate prediction of protein structures and interactions using a three-track neural network. Science (New York, N.Y.).

