# Supplementary material for: Designing a multi-epitope vaccine to provoke the robust immune response against influenza A H7N9
Source: Sci Rep. 2021 Dec 29;11:24485. doi: 10.1038/s41598-021-03932-2 (PMC8716528; doi:10.1038/s41598-021-03932-2)
Supplement: Supplementary file 1 — Supplementary Information. [file 41598_2021_3932_MOESM1_ESM.pdf]

Designing a multi-epitope vaccine to provoke the robust immune response against Influenza A H7N9

Hossein Tarrahimofrad[1], Somayyeh Rahimnahal[2], Javad Zamani[1], Ehsan Jahangirian[1], Saeed Aminzadeh[1]*

[1] Bioprocess Engineering Group, Institute of Industrial and Environmental Biotechnology, National Institute of Genetic Engineering and Biotechnology (NIGEB), Tehran, Iran

[2] Department of Animal Science, Faculty of Agriculture, Ilam University, Ilam, Iran

***Corresponding Author; Saeed Aminzadeh**

E-mail: aminzade@nigeb.ac.ir

Table S1. Predicted discontinuous B-cell epitopes from HA and NA.

**No.**	**Residues**	**Number of residues**	**Score**
HA			
1	D1, K2, I3, C4, A9, V10, S11, N12, G13, K15, T18, L19, T20, E21, G23, E25, V27, **K312**, **N313**, **V314**, **P315**, **E316**, G322, L323, F324, G325, A326, I327, A328, G329, F330, I331, E332, **N333**, **G334**, **W335**, **E336**, **G337**, **L338**, **I339**, **D340**, **G341**, **F345**, **R346**, **H347**, **Q348**, **N349**, **A350**, **Q351**, **G352**, **E353**, **G354**, **T355**, **A356**, **A357**, **D437**, **K438**, **Y440**, **E441**, **R442**, V443, K444, R445, Q446, L447, R448, **E449**, **N450**, **A451**, **E452**, **D454**, **G455**, **T456**, G457, C458, F459, E460, I461, F462, H463, K464, C465, D466, D467, D468, C469, M470, A471, S472, **I473**, **R474**, **N475**, **N476**, **T477**, **Y478**, **D479**, **H480**, **S481**, **K482**, **Y483**, **R484**, **E485**, **E486**, **A487**, **M488**, **Q489**, **N490**	107	0.731
2	**R47**, **P64**, **D67**, **Q68**, **F69**, **L70**, **E71**, **F72**, **S73**, **A74**, **D75**, **C87**, **Y88**, **P89**, **G90**, **G106**, **G107**, **D109**, **E111**, **A112**, **M113**, **G114**, **F115**, **T116**, **Y117**, **S118**, **G119**, **I120**, **R121**, **T122**, **N123**, **G124**, **A125**, **T126**, **S127**, **A128**, **C129**, **R130**, **R131**, **S132**, **G133**, **S134**, **S135**, **F136**, **Y137**, **E139**, L143, S145, **N146**, **T147**, **D148**, **N149**, **A150**, **A151**, **F152**, **P153**, **Q154**, **M155**, **T156**, **K157**, **S158**, **Y159**, **K160**, **N161**, **T162**, **R163**, **K164**, N165, P166, S176, V177, **S178**, **T179**, **A180**, **E181**, **Q182**, **T183**, **K184**, **L185**, **G187**, **S188**, **G189**, N190, K191, L192, V193, T194, V195, G196, S197, S198, **N199**, **Y200**, **Q202**, **S203**, **F204**, **V205**, **P206**, **S207**, **P208**, **G209**, **A210**, **R211**, **P212**, **Q213**, **V214**, **N215**, **G216**, **L217**, **S218**, **G219**, **R220**, L228, N229, P230, N231, D232, T233, **V234**, **T235**, **F236**, **S237**, **F238**, **N239**, **G240**, **D246**, **R247**, **F250**, **R252**	129	0.688
3	V394, E395, K396, Q397, G399, N400, V401, W404	8	0.665
4	D407, S408, E411	3	0.566
5	**N37**, **I38**, **P39**, **R40**, **S43**, **K44**, **G45**, **K46**, **V262**, **Q263**, **V264**, **D265**, **A266**, **D267**, C268, E269, G276	17	0.518
**NA**			
1	N104, V106, R107, I108, G109, E110, D111, S112, D113, V114, L115, V116, S135, Q136, G137, **T138**, **T139**, **I140**, **R141**, **G142**, **K143**, **H144**, **S145**, **N146**, **G147**, **T148**, **I149**, **H150**, **S165**, **P166**, **P167**, **T168**, **V169**, **N170**, **S171**, , S372, **L399**, **N400**, **T401**, **D402**, G429, R430, P431, K432, E433, D434, K435, V436, W437, W438, W456, N457, W458, P459, D460, G461, A462, K463, I464, E465, Y466, F467	63	0.684
2	**R82**, **E83**, **F84**, **N85**, **N86**, **L87**, **T88**, **K89**, **G90**, **L91**, C92, T93, I94, N95, S96, P126, D127, E128, **G177**, W178, D185, G186, R187, A188, S195, G196, P197, N198, N199, N200, A201, S202, V204, W206, Y207, N208, R210, P211, V212, T213, E214, I215, N216, T217, W218, A219, R220, N221, I222, L223, Q233, N234, G235, V236, D243, G244, S245, **A246**, **T247**, **G248**, **P249**, **A250**, **E251**, R253, K258, E259, G260, K261, I262, L263, K264, W265, E266, P267, L268, T269, G270, T271, A272, K273, Q284, A285, D293, N294, W295, Q296, G297, S298, N299, **D306**, **P307**, **V308**, **A309**, **M310**, **T311**, I317, C318, S319, P320, V321, L322, **N325**, **P326**, **R327**, **P328**, **N329**, **D330**, **P331**, **T332**, **V333**, **G335**, **K336**, **C337**, **N338**, **D339**, **P340**, **Y341**, **P342**, **G343**, **N344**, **N345**, **N346**, **N347**, D356, G357, G358, N359, T360, **S367**, **I368**, **A369**, **S370**, K378, V379, P380, **N381**, **A382**, **L383**, **T384**, **D385**, **D386**, **R387**, **S388**, **K389**, **P390**, **T391**, **Q392**, G394, T396, A413, E414, G415, E416, C417, Y418, S449, T450, E451, F452, L453, G454, Q455	162	0.665
3	**S153**, **Q154**, **Y155**, R156	4	0.59

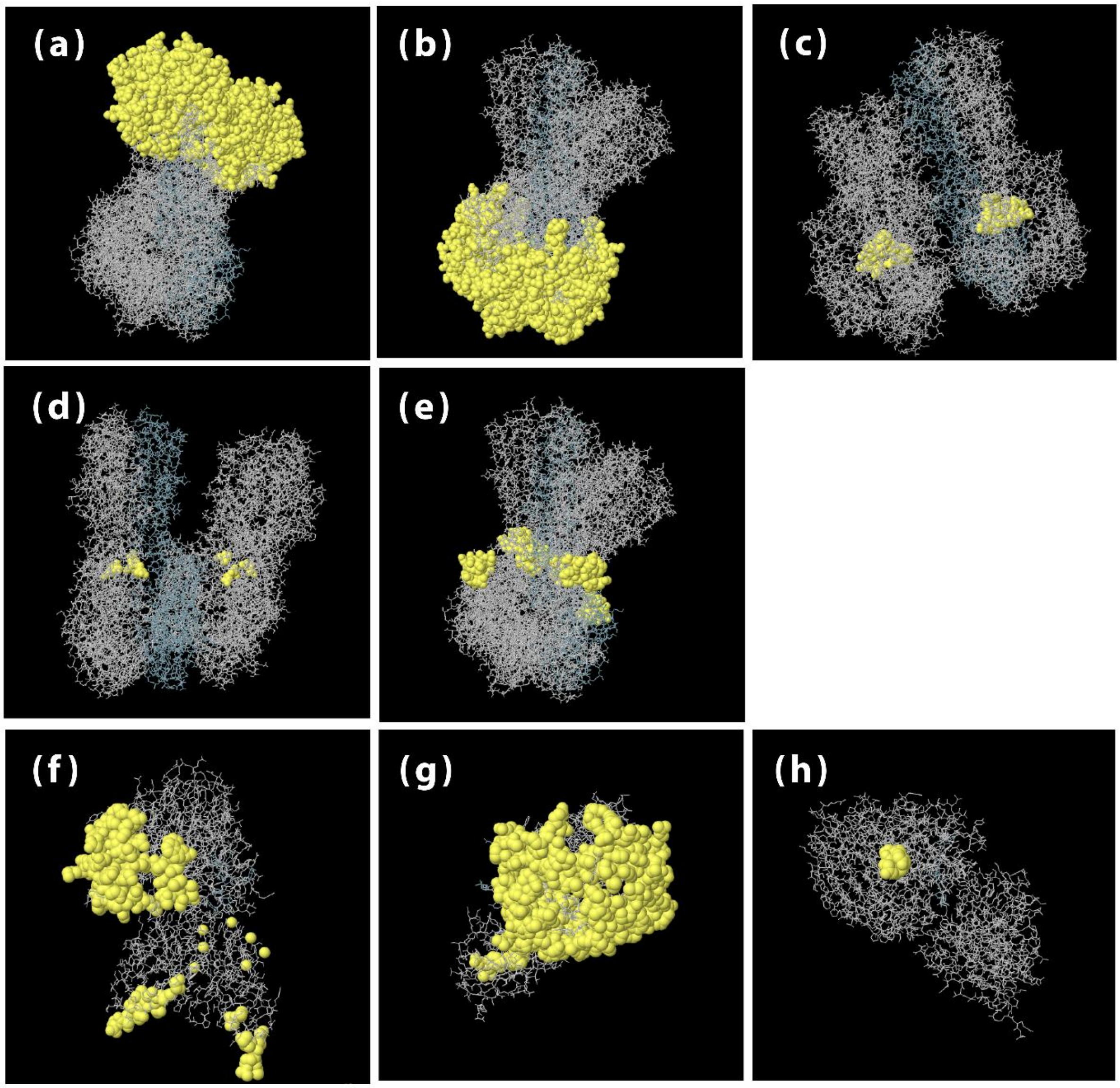

Figure S1. Visualized discontinuous B-cell epitopes from HA and NA based on Table S1 presented information. Visualized snapshots of discontinuous B-cell epitopes related to HA from (a) to (e) and discontinuous B-cell epitopes related to NA from (f) to (h) are compatible with numbers from rows 1 to 5 for HA and from rows 1 to 3 for NA in Table S1.

Supplementary files:

Table S2. Prediction CTL epitopes of HA and NA proteins.

Pos	HLA	Peptide	1-log50k (aff)	Affinity (nM)	% Rank	Bind Level	Antigenicity
**HA**							
**195**	HLA-A0101	STAEQTKLY	0.502	217.68	0.25	<= SB	0.1782
**535**	HLA-A0201	ILLAIVMGL	0.798	8.88	0.09	<= SB	0.8032
**536**	HLA-A0202	LLAIVMGLV	0.806	8.20	0.17	<= SB	0.8049
**267**	HLA-A0203	FLRGKSMGI	0.881	3.61	0.04	<= SB	1.0137
**323**	HLA-A0205	LLATGMKNV	0.384	781.26	0.17	<= SB	0.8738
**403**	HLA-A0206	LIDNEFNEV	0.808	7.98	0.12	<= SB	0.4646
**540**	HLA-A0207	VMGLVFICV	0.200	5713.19	0.15	<= SB	0.6528
**243**	HLA-A0211	LMLNPNDTV	0.915	2.52	0.05	<= SB	0.7966
**243**	HLA-A0212	LMLNPNDTV	0.827	6.53	0.08	<= SB	0.7966
**243**	HLA-A0216	LMLNPNDTV	0.824	6.69	0.07	<= SB	0.7966
**535**	HLA-A0217	ILLAIVMGL	0.597	78.30	0.12	<= SB	0.8032
**540**	HLA-A0219	VMGLVFICV	0.809	7.92	0.06	<= SB	0.6528
**267**	HLA-A0250	FLRGKSMGI	0.833	6.10	0.08	<= SB	1.0137
**321**	HLA-A0301	SLLLATGMK	0.663	38.40	0.17	<= SB	0.8143
**150**	HLA-A1101	GSSFYAEMK	0.719	20.85	0.12	<= SB	0.5085
**519**	HLA-A2301	GYKDVILWF	0.708	23.68	0.09	<= SB	-0.0897
**153**	HLA-A2402	FYAEMKWLL	0.670	35.56	0.06	<= SB	0.6900
**519**	HLA-A2403	GYKDVILWF	0.848	5.18	0.06	<= SB	-0.0897
**259**	HLA-A2501	FIAPDRASF	0.421	524.24	0.06	<= SB	0.7547
**195**	HLA-A2601	STAEQTKLY	0.759	13.62	0.03	<= SB	0.1782
**195**	HLA-A2602	STAEQTKLY	0.814	7.45	0.03	<= SB	0.1782

**251**	HLA-A2603	VTFSFNGAF	0.455	365.74	0.05	<= SB	1.0613
**526**	HLA-A2902	WFSFGASCF	0.643	47.63	0.40	<= SB	1.7382
**513**	HLA-A3001	PVKLSSGYK	0.610	68.36	0.40	<= SB	0.6758
**308**	HLA-A3002	RAVGKCPRY	0.630	54.72	0.17	<= SB	1.0469
**172**	HLA-A3101	MTKSYKNTR	0.838	5.77	0.03	<= SB	0.6705
**521**	HLA-A3201	KDVILWFSF	0.714	22.05	0.05	<= SB	0.3832
**315**	HLA-A3207	RYVKQRSLL	0.637	50.87	0.12	<= SB	-0.4114
**151**	HLA-A3215	SSFYAEMKW	0.469	311.58	0.08	<= SB	1.0372
**172**	HLA-A3301	MTKSYKNTR	0.715	21.74	0.08	<= SB	0.6705
**294**	HLA-A6601	GTIISNLPF	0.291	2155.15	0.06	<= SB	0.5474
**89**	HLA-A6801	FSADLIIER	0.842	5.53	0.02	<= SB	0.1139
**42**	HLA-A6802	EVVNATETV	0.879	3.69	0.03	<= SB	0.8466
**493**	HLA-A6823	NTYDHSKYR	0.630	54.69	0.12	<= SB	-0.0574
**428**	HLA-A6901	EVWSYNAEL	0.867	4.23	0.01	<= SB	0.0106
**195**	HLA-A8001	STAEQTKLY	0.576	98.24	0.07	<= SB	0.1782
**228**	HLA-B0702	RPQVNGLSG	0.490	249.26	0.80	<= WB	0.7145
**316**	HLA-B0801	YVKQRSLLL	0.721	20.55	0.06	<= SB	0.9156
**316**	HLA-B0802	YVKQRSLLL	0.263	2916.65	0.02	<= SB	0.9156
**316**	HLA-B0803	YVKQRSLLL	0.394	704.80	0.01	<= SB	0.9156
**265**	HLA-B1402	ASFLRGKSM	0.467	320.84	0.03	<= SB	0.5780
**537**	HLA-B1501	LAIVMGLVF	0.774	11.58	0.07	<= SB	0.9184
**354**	HLA-B1502	GLIDGWYGF	0.565	110.90	0.06	<= SB	0.2489
**145**	HLA-B1503	ACRRSGSSF	0.809	7.87	0.09	<= SB	0.4887
**191**	HLA-B1509	HHSVSTAEQ	0.335	1333.21	0.20	<= SB	0.5952
**320**	HLA-B1517	RSLLLATGM	0.924	2.29	0.03	<= SB	0.6310

**352**	HLA-B1801	WEGLIDGWY	0.566	110.00	0.30	<= SB	-0.9482
**236**	HLA-B2705	GRIDFHWLM	0.629	55.62	0.25	<= SB	2.2522
**487**	HLA-B3501	MASIRNNTY	0.782	10.60	0.06	<= SB	0.6577
**259**	HLA-B3503	FIAPDRASF	0.261	2974.20	0.12	<= SB	0.7547
**153**	HLA-B3801	FYAEMKWLL	0.361	1002.01	0.30	<= SB	0.6900
**432**	HLA-B3901	YNAELLVAM	0.532	157.32	0.25	<= SB	0.5395
**87**	HLA-B4001	LEFSADLII	0.705	24.25	0.12	<= SB	0.4113
**440**	HLA-B4002	MENQHTIDL	0.616	63.70	0.20	<= SB	1.0044
**527**	HLA-B4013	FSFGASCFI	0.487	258.45	0.09	<= SB	1.3232
**182**	HLA-B4201	SPALIVWGI	0.583	91.32	0.12	<= SB	0.6161
**440**	HLA-B4402	MENQHTIDL	0.609	68.89	0.15	<= SB	1.0044
**155**	HLA-B4403	AEMKWLLSN	0.525	169.92	0.30	<= SB	0.3105
**155**	HLA-B4501	AEMKWLLSN	0.560	116.98	0.25	<= SB	0.3105
**537**	HLA-B4601	LAIVMGLVF	0.407	614.34	0.06	<= SB	0.9184
**2**	HLA-B4801	TQILVFALI	0.282	2367.29	0.25	<= SB	0.1934
**438**	HLA-B5101	VAMENQHTI	0.461	341.93	0.07	<= SB	0.9184
**537**	HLA-B5301	LAIVMGLVF	0.575	99.43	0.17	<= SB	0.9184
**253**	HLA-B5401	FSFNGAFIA	0.634	52.38	0.17	<= SB	0.8681
**151**	HLA-B5701	SSFYAEMKW	0.836	5.89	0.01	<= SB	1.0372
**234**	HLA-B5801	LSGRIDFHW	0.930	2.14	0.01	<= SB	2.2921
**151**	HLA-B5802	SSFYAEMKW	0.171	7835.55	0.06	<= SB	1.0372
**147**	HLA-B7301	RRSGSSFYA	0.289	2180.93	0.07	<= SB	0.3339
**300**	HLA-B8301	LPFQNIDSR	0.385	775.48	0.09	<= SB	1.1190
**527**	HLA-C0303	FSFGASCFI	0.797	9.02	0.07	<= SB	1.3232
**526**	HLA-C0401	WFSFGASCF	0.361	1007.64	0.01	<= SB	1.7382

**161**	HLA-C0501	LSNTDNAAF	0.641	48.60	0.08	<= SB	0.8482
**314**	HLA-C0602	PRYVKQRSL	0.549	131.73	0.08	<= SB	-0.1159
**316**	HLA-C0701	YVKQRSLLL	0.590	84.44	0.04	<= SB	0.9156
**153**	HLA-C0702	FYAEMKWLL	0.531	160.12	0.05	<= SB	0.6900
**403**	HLA-C0802	LIDNEFNEV	0.340	1256.78	0.10	<= SB	0.4646
**438**	HLA-C1203	VAMENQHTI	0.769	12.18	0.03	<= SB	0.9818
**168**	HLA-C1402	AFPQMTKSY	0.688	29.35	0.10	<= SB	0.1453
**430**	HLA-C1502	WSYNAELLV	0.697	26.49	0.01	<= SB	0.4962
**NA**							
**87**	HLA-A0101	CTINSWHIY	0.517	185.25	0.25	<= SB	0.4951
**349**	HLA-A0201	YLDGANTWL	0.797	8.98	0.10	<= SB	-0.5304
**349**	HLA-A0202	YLDGANTWL	0.828	6.46	0.12	<= SB	-0.5304
**24**	HLA-A0203	GMANLGLNI	0.773	11.64	0.40	<= SB	1.2411
**109**	HLA-A0205	VLVTREPYV	0.529	162.81	0.03	<= SB	0.9493
**349**	HLA-A0206	YLDGANTWL	0.694	27.35	0.50	<= SB	-0.5304
**349**	HLA-A0207	YLDGANTWL	0.203	5580.61	0.12	<= SB	-0.5304
**349**	HLA-A0211	YLDGANTWL	0.865	4.29	0.15	<= SB	-0.5304
**305**	HLA-A0212	AMTHTSQYI	0.808	8.02	0.10	<= SB	0.3111
**448**	HLA-A0216	FLGQWNWPD	0.867	4.19	0.03	<= SB	0.8316
**441**	HLA-A0217	SMCSSTEFL	0.569	105.91	0.17	<= SB	0.0551
**349**	HLA-A0219	YLDGANTWL	0.757	13.79	0.10	<= SB	-0.5304
**349**	HLA-A0250	YLDGANTWL	0.807	8.05	0.12	<= SB	-0.5304
**249**	HLA-A0301	RIYYFKEGK	0.733	17.92	0.06	<= SB	-0.1232
**249**	HLA-A1101	RIYYFKEGK	0.680	31.86	0.25	<= SB	-0.1232
**348**	HLA-A2301	SYLDGANTW	0.591	83.56	0.30	<= SB	-0.1537

**164**	HLA-A2402	VYNSRVECI	0.557	120.79	0.25	<= SB	1.4709
**348**	HLA-A2403	SYLDGANTW	0.899	2.99	0.02	<= SB	-0.1537
**304**	HLA-A2501	HTSQYICSP	0.274	2566.87	0.30	<= SB	-0.1098
**87**	HLA-A2601	CTINSWHIY	0.719	20.83	0.04	<= SB	0.4951
**87**	HLA-A2602	CTINSWHIY	0.826	6.60	0.03	<= SB	0.4951
**434**	HLA-A2603	WTSNSIVSM	0.408	602.34	0.09	<= SB	0.6097
**399**	HLA-A2902	GYSGSFMDY	0.729	18.81	0.15	<= SB	0.7338
**183**	HLA-A3001	KSRMSICIS	0.783	10.50	0.08	<= SB	0.7017
**87**	HLA-A3002	CTINSWHIY	0.711	22.70	0.04	<= SB	0.4951
**197**	HLA-A3101	ASAVVWYNR	0.841	5.57	0.03	<= SB	0.9375
**431**	HLA-A3201	KVWWTSNSI	0.865	4.30	0.01	<= SB	0.2327
**348**	HLA-A3207	SYLDGANTW	0.640	49.10	0.12	<= SB	-0.1537
**148**	HLA-A3215	SQYRALISW	0.400	659.35	0.17	<= SB	0.2439
**212**	HLA-A3301	NTWARNILR	0.832	6.16	0.01	<= SB	-1.4021
**142**	HLA-A6601	GTIHDRSQY	0.268	2741.14	0.15	<= SB	1.5360
**105**	HLA-A6801	ESSDVLVTR	0.846	5.27	0.01	<= SB	0.7419
**309**	HLA-A6802	TSQYICSPV	0.792	9.51	0.15	<= SB	0.0851
**400**	HLA-A6823	YSGSFMDYW	0.586	88.34	0.17	<= SB	0.0400
**412**	HLA-A6901	DCYRACFYV	0.730	18.53	0.09	<= SB	0.6386
**328**	HLA-A8001	NIGKCNDPY	0.475	294.17	0.15	<= SB	-0.2737
**192**	HLA-B0702	GPNNNASAV	0.629	55.31	0.30	<= SB	0.3885
**370**	HLA-B0801	EMLKVPNAL	0.415	558.83	1.00	<= WB	-0.2027
**245**	HLA-B0802	PADTRIYYF	0.201	5659.11	0.08	<= SB	0.2964
**150**	HLA-B0803	YRALISWPL	0.195	6087.55	0.17	<= SB	-0.0645
**148**	HLA-B1402	SQYRALISW	0.365	961.52	0.17	<= SB	0.2439

**440**	HLA-B1501	VSMCSSTEF	0.742	16.39	0.12	<= SB	0.2568
**392**	HLA-B1502	VLNADWSGY	0.606	71.33	0.03	<= SB	0.6849
**440**	HLA-B1503	VSMCSSTEF	0.784	10.40	0.15	<= SB	0.2568
**310**	HLA-B1509	SQYICSPVL	0.468	314.95	0.05	<= SB	-0.1783
**361**	HLA-B1517	ISTASRSGY	0.876	3.81	0.09	<= SB	1.0412
**446**	HLA-B1801	TEFLGQWNW	0.706	24.02	0.09	<= SB	0.4802
**150**	HLA-B2705	YRALISWPL	0.796	9.13	0.01	<= SB	-0.0645
**150**	HLA-B2720	YRALISWPL	0.909	2.66	0.01	<= SB	-0.0645
**454**	HLA-B3501	WPDGAKIEY	0.842	5.52	0.02	<= SB	0.3169
**251**	HLA-B3503	YYFKEGKIL	0.129	12354.51	0.70	<= WB	0.5783
**146**	HLA-B3801	DRSQYRALI	0.418	544.74	0.17	<= SB	0.2429
**150**	HLA-B3901	YRALISWPL	0.904	2.83	0.01	<= SB	-0.0645
**261**	HLA-B4001	WESLTGTAK	0.445	403.48	0.80	<= WB	0.8383
**369**	HLA-B4002	YEMLKVPNA	0.687	29.44	0.06	<= SB	-0.0588
**310**	HLA-B4013	SQYICSPVL	0.562	114.15	0.04	<= SB	-0.1783
**296**	HLA-B4201	RPVIQIDPV	0.506	209.57	0.25	<= SB	0.8219
**446**	HLA-B4402	TEFLGQWNW	0.816	7.33	0.01	<= SB	0.4802
**446**	HLA-B4403	TEFLGQWNW	0.607	70.28	0.12	<= SB	0.4802
**369**	HLA-B4501	YEMLKVPNA	0.643	47.51	0.07	<= SB	-0.0588
**440**	HLA-B4601	VSMCSSTEF	0.523	175.00	0.01	<= SB	0.2568
**310**	HLA-B4801	SQYICSPVL	0.546	135.66	0.01	<= SB	-0.1783
**156**	HLA-B5101	WPLSSPPTV	0.597	78.60	0.01	<= SB	0.1944
**206**	HLA-B5301	RPVAEINTW	0.631	54.20	0.12	<= SB	-0.7724
**156**	HLA-B5401	WPLSSPPTV	0.525	170.12	0.40	<= SB	0.1944
**84**	HLA-B5701	KGLCTINSW	0.696	26.79	0.07	<= SB	0.5352

**400**	HLA-B5801	YSGSFMDYW	0.822	6.86	0.04	<= SB	0.0400
**444**	HLA-B5802	SSTEFLGQW	0.146	10297.52	0.25	<= SB	-0.5175
**150**	HLA-B7301	YRALISWPL	0.302	1909.77	0.06	<= SB	-0.0645
**244**	HLA-B8301	GPADTRIYY	0.388	747.44	0.08	<= SB	0.7014
**127**	HLA-C0303	YALSQGTTI	0.855	4.79	0.03	<= SB	0.5916
**349**	HLA-C0401	YLDGANTWL	0.306	1818.15	0.03	<= SB	-0.5304
**349**	HLA-C0501	YLDGANTWL	0.803	8.39	0.01	<= SB	-0.5304
**251**	HLA-C0602	YYFKEGKIL	0.495	235.14	0.15	<= SB	0.5783
**251**	HLA-C0701	YYFKEGKIL	0.504	213.38	0.10	<= SB	0.5783
**414**	HLA-C0702	YRACFYVEL	0.583	91.50	0.03	<= SB	0.8895
**378**	HLA-C0802	LTDDRSKPI	0.409	598.79	0.04	<= SB	0.4508
**95**	HLA-C1203	YGKDNAVRI	0.647	45.63	0.15	<= SB	-0.6476
**251**	HLA-C1402	YYFKEGKIL	0.806	8.12	0.01	<= SB	0.5783
**366**	HLA-C1502	RSGYEMLKV	0.668	36.13	0.02	<= SB	0.6568

Table S3. Prediction HTL epitopes of HA and NA proteins

Allele	Pos.	Peptide	1-log50k(aff)	Affinity (nM)	% Rank	Relia	Bind Level	Antigenicity
**HA**								
DRB1_0101	240	DFHWLMLNPNDTVTF	0.8352	5.9	0.90	0.51	SB	1.4982
DRB1_0103	452	SEMDKLYERVKRQLR	0.2757	2532.7	1.50	0.38	SB	0.1100
DRB1_0301	507	QNRIQIDPVKLSSGY	0.7479	15.3	0.50	0.92	SB	0.7553
DRB1_0401	154	FYAEMKWLLSNTDNA	0.6983	26.2	0.80	0.42	SB	0.4750

DRB1_0402	523	DVILWFSFGASCFIL	0.5309	160.0	0.03	0.37	SB	0.7926
DRB1_0403	539	AIVMGLVFICVKNGN	0.4535	369.7	0.06	0.21	SB	1.3687
DRB1_0404	7	VFALIAIIPTNADKI	0.6865	29.7	1.50	0.60	SB	0.4662
DRB1_0405	397	KTNQQFELIDNEFNE	0.7120	22.6	0.50	0.88	SB	0.9297
DRB1_0701	262	APDRASFLRGKSMGI	0.8190	7.1	0.50	0.96	SB	0.4938
DRB1_0801	130	AMGFTYSGIRTNGAT	0.6114	67.0	3.00	0.65	SB	0.2620
DRB1_0802	7	VFALIAIIPTNADKI	0.7370	17.2	0.04	0.35	SB	0.4662
DRB1_0901	264	DRASFLRGKSMGIQS	0.7592	13.5	0.40	0.92	SB	0.5249
DRB1_1001	7	VFALIAIIPTNADKI	0.8022	8.5	0.60	0.52	SB	0.4662
DRB1_1101	456	KLYERVKRQLRENAE	0.6684	36.1	4.00	0.74	WB	0.0174
DRB1_1201	292	HSGGTIISNLPFQNI	0.6202	60.9	1.70	0.64	SB	0.4323
DRB1_1301	455	DKLYERVKRQLRENA	0.8280	6.4	1.20	0.49	SB	-0.0546
DRB1_1302	241	FHWLMLNPNDTVTFS	0.8059	8.2	1.50	0.49	SB	1.1847
DRB1_1501	424	RDSITEVWSYNAELL	0.7384	16.9	1.20	0.64	SB	-0.1026
DRB1_1602	313	KCPRYVKQRSLLLAT	0.7368	17.3	0.40	0.84	SB	0.5888
DRB3_0101	256	FNGAFIAPDRASFLR	0.7369	17.2	0.90	0.81	SB	-0.0276
DRB3_0202	241	FHWLMLNPNDTVTFS	0.8026	8.5	0.60	0.61	SB	1.1847
DRB3_0301	241	FHWLMLNPNDTVTFS	0.8466	5.3	0.70	0.63	SB	1.1847
DRB4_0101	435	AELLVAMENQHTIDL	0.6748	33.7	1.50	0.57	SB	0.8062
DRB4_0103	456	KLYERVKRQLRENAE	0.7669	12.5	2.50	0.34	WB	0.0174
DRB5_0101	318	VKQRSLLLATGMKNV	0.7548	14.2	2.50	0.50	SB	1.0222
DPA10103-DPB10301	318	VKQRSLLLATGMKNV	0.6291	55.3	1.00	0.50	SB	1.0222
DPA10103-DPB10401	525	ILWFSFGASCFILLA	0.7475	15.4	0.50	0.72	SB	1.0849

DPA10103-DPB10402	346	AGFIENGWEGLIDGW	0.3498	1136.2	0.90	0.21	SB	-0.2073
DPA10103-DPB10601	2	NTQILVFALIAIIPT	0.7910	9.6	3.00	0.57	SB	0.5484
DPA10201-DPB10101	524	VILWFSFGASCFILL	0.6300	54.8	1.10	0.58	SB	0.9305
DPA10201-DPB10501	318	VKQRSLLLATGMKNV	0.6462	46.0	0.40	0.49	SB	1.0222
DPA10201-DPB11401	316	RYVKQRSLLLATGMK	0.5016	219.8	1.40	0.27	SB	0.4843
DPA10301-DPB10402	86	QFLEFSADLIIERRE	0.6107	67.5	4.00	0.29	WB	0.9921
DPA10103-DPB10201	524	VILWFSFGASCFILL	0.7106	22.9	0.80	0.37	SB	0.9305
DQA10101-DQB10501	84	CDQFLEFSADLIIER	0.6698	35.6	0.60	0.34	SB	0.1805
DQA10102-DQB10501	8	FALIAIIPTNADKIC	0.8321	6.1	0.20	0.52	SB	0.8252
DQA10102-DQB10502	86	QFLEFSADLIIERRE	0.5798	94.3	1.10	0.35	SB	0.9921
DQA10102-DQB10602	194	SVSTAEQTKLYGSGN	0.5910	83.6	1.80	0.89	SB	0.1206
DQA10103-DQB10603	249	NDTVTFSFNGAFIAP	0.5575	120.0	0.40	0.40	SB	0.5069

DQA10104-DQB10503	355	GLIDGWYGFRHQNAQ	0.4996	224.6	0.80	0.49	SB	0.6786
DQA10201-DQB10202	424	RDSITEVWSYNAELL	0.5180	184.0	0.60	0.34	SB	-0.1026
DQA10201-DQB10301	136	SGIRTNGATSACRRS	0.7710	11.9	2.50	0.32	WB	0.0500
DQA10201-DQB10303	216	SNYQQSFVPSPGARP	0.5800	94.1	5.00	0.21	WB	0.4629
DQA10201-DQB10402	130	AMGFTYSGIRTNGAT	0.6976	26.4	0.40	0.34	SB	0.2620
DQA10301-DQB10301	185	ALIVWGIHHSVSTAE	0.6565	41.1	0.80	0.37	SB	0.3415
DQA10301-DQB10302	250	DTVTFSFNGAFIAPD	0.5297	162.1	0.60	0.42	SB	0.4568
DQA10303-DQB10402	356	LIDGWYGFRHQNAQG	0.5151	189.9	1.20	0.38	SB	0.5347
DQA10401-DQB10402	83	QCDQFLEFSADLIIE	0.4941	238.2	1.50	0.43	SB	0.2344
DQA10501-DQB10201	395	IEKTNQQFELIDNEF	0.5954	79.6	1.40	0.73	SB	1.1539
DQA10501-DQB10301	288	GDCYHSGGTIISNLP	0.7425	16.2	0.17	0.54	SB	0.0010
DQA10501-DQB10302	250	DTVTFSFNGAFIAPD	0.5453	136.9	1.10	0.56	SB	0.4568

DQA10501-DQB10303	187	IVWGIHHSVSTAEQT	0.5715	103.1	3.00	0.28	WB	0.6305
DQA10501-DQB10402	130	AMGFTYSGIRTNGAT	0.7394	16.8	0.50	0.48	SB	0.2620
DQA10601-DQB10402	184	PALIVWGIHHSVSTA	0.4931	241.0	3.00	0.46	WB	0.3835
**NA**								
DRB1_0101	367	RSGYEMLKVPNALTD	0.8565	4.7	0.50	0.87	SB	0.1873
DRB1_0103	417	ACFYVELIRGRPKED	0.2966	2020.6	0.70	0.57	SB	0.6882
DRB1_0301	297	RPVIQIDPVAMTHTS	0.7758	11.3	0.25	0.90	SB	1.0194
DRB1_0401	59	NNYYNETNITNIQME	0.6766	33.1	1.20	0.85	SB	1.0803
DRB1_0402	21	AVLIGMANLGLNIGL	0.4306	474.0	1.70	0.49	SB	1.4582
DRB1_0403	21	AVLIGMANLGLNIGL	0.4029	639.4	1.60	0.19	SB	1.4582
DRB1_0404	20	IAVLIGMANLGLNIG	0.6881	29.2	1.50	0.61	SB	1.4039
DRB1_0405	145	IHDRSQYRALISWPL	0.6795	32.1	1.10	0.83	SB	0.5792
DRB1_0701	2	NPNQKILCTSATAII	0.7756	11.3	1.20	0.49	SB	0.5567
DRB1_0801	198	ASAVVWYNRRPVAEI	0.6647	37.6	1.00	0.35	SB	0.6754
DRB1_0802	197	NASAVVWYNRRPVAE	0.6143	64.9	1.00	0.34	SB	0.6669
DRB1_0901	124	ECRFYALSQGTTIRG	0.8110	7.7	0.07	0.83	SB	0.5557
DRB1_1001	346	KGFSYLDGANTWLGR	0.7509	14.8	1.90	0.80	SB	0.2596
DRB1_1101	126	RFYALSQGTTIRGKH	0.6287	55.6	6.00	0.44	WB	0.9183
DRB1_1201	437	SNSIVSMCSSTEFLG	0.5481	132.8	5.00	0.47	WB	-0.0941
DRB1_1301	200	AVVWYNRRPVAEINT	0.6821	31.2	9.00	0.34	WB	0.5862
DRB1_1302	23	IGMANLGLNIGLHLK	0.7992	8.8	1.60	0.71	SB	1.9099
DRB1_1501	150	QYRALISWPLSSPPT	0.7265	19.3	1.50	0.64	SB	0.0857

DRB1_1602	145	IHDRSQYRALISWPL	0.7255	19.5	0.50	0.66	SB	0.5792
DRB3_0101	298	PVIQIDPVAMTHTSQ	0.6832	30.8	1.80	0.94	SB	1.1009
DRB3_0202	431	DKVWWTSNSIVSMCS	0.7309	18.4	1.50	0.55	SB	0.0422
DRB3_0301	6	KILCTSATAIIIGAI	0.8474	5.2	0.70	0.48	SB	0.6940
DRB4_0101	297	RPVIQIDPVAMTHTS	0.6825	31.0	1.30	0.38	SB	1.0194
DRB4_0103	21	AVLIGMANLGLNIGL	0.6584	40.3	9.50	0.57	WB	1.4582
DRB5_0101	416	RACFYVELIRGRPKE	0.7665	12.5	1.90	0.62	SB	0.8598
DPA10103-DPB10301	146	HDRSQYRALISWPLS	0.5787	95.5	1.70	0.38	SB	0.3692
DPA10103-DPB10401	440	IVSMCSSTEFLGQWN	0.6531	42.7	1.90	0.75	SB	-0.1086
DPA10301-DPB10402	248	DTRIYYFKEGKILKW	0.3352	1329.9	1.80	0.31	SB	0.0360
DPA10103-DPB10601	414	CYRACFYVELIRGRP	0.8201	7.0	1.90	0.40	SB	0.8122
DPA10201-DPB10101	413	DCYRACFYVELIRGR	0.5948	80.1	2.50	0.22	SB	0.5842
DPA10201-DPB10501	248	DTRIYYFKEGKILKW	0.5764	97.9	1.30	0.38	SB	0.0360
DPA10201-DPB11401	145	IHDRSQYRALISWPL	0.3658	954.9	8.50	0.35	WB	0.5792
DPA10301-DPB10402	414	CYRACFYVELIRGRP	0.6458	46.2	2.50	0.51	WB	0.8122
DPA10103-DPB10201	248	DTRIYYFKEGKILKW	0.5934	81.4	5.50	0.29	WB	0.0360

DQA10101-DQB10501	411	EGDCYRACFYVELIR	0.5997	76.0	1.70	0.50	SB	0.1877
DQA10102-DQB10501	21	AVLIGMANLGLNIGL	0.7790	10.9	1.30	0.57	SB	1.4582
DQA10102-DQB10502	400	GYSGSFMDYWAEGDC	0.5854	88.7	1.00	0.31	SB	0.0726
DQA10102-DQB10602	6	KILCTSATAIIIGAI	0.5904	84.1	1.80	0.44	SB	0.6940
DQA10103-DQB10603	8	LCTSATAIIIGAIAV	0.5389	146.9	0.60	0.23	SB	0.5690
DQA10104-DQB10503	402	SGSFMDYWAEGDCYR	0.4933	240.3	0.90	0.31	SB	-0.1237
DQA10201-DQB10202	306	AMTHTSQYICSPVLT	0.3645	968.3	7.50	0.32	WB	0.2056
DQA10201-DQB10301	11	SATAIIIGAIAVLIG	0.8081	8.0	1.40	0.25	SB	0.6592
DQA10201-DQB10303	435	WTSNSIVSMCSSTEF	0.6615	39.0	1.40	0.23	SB	0.2858
DQA10201-DQB10402	85	KGLCTINSWHIYGKD	0.6208	60.5	1.40	0.44	SB	0.7974
DQA10301-DQB10301	394	LNADWSGYSGSFMDY	0.5951	79.9	2.50	0.22	WB	0.8200
DQA10301-DQB10302	62	YNETNITNIQMEERT	0.4713	304.9	1.90	0.74	SB	1.4338

DQA10303-DQB10402	356	DQA10303-DQB10402	0.4864	259.1	1.60	0.23	SB	0.8316
DQA10401-DQB10402	61	YYNETNITNIQMEER	0.5090	202.9	1.20	0.50	SB	1.4272
DQA10501-DQB10201	401	YSGSFMDYWAEGDCY	0.5216	177.1	4.50	0.39	WB	-0.0063
DQA10501-DQB10301	356	TWLGRTISTASRSGY	0.5073	206.7	9.50	0.19	WB	0.8316
DQA10501-DQB10302	397	DWSGYSGSFMDYWAE	0.5518	127.6	0.90	0.60	SB	0.2654
DQA10501-DQB10303	7	ILCTSATAIIIGAIA	0.5927	82.0	1.60	0.14	SB	0.6886
DQA10501-DQB10402	149	SQYRALISWPLSSPP	0.7044	24.5	0.90	0.51	SB	0.0011
DQA10601-DQB10402	84	TKGLCTINSWHIYGK	0.5838	90.3	0.80	0.35	SB	0.6794

Table S4. Detailed population coverage of HA and NA proteins. Projected population coverage average number of epitope hits / HLA combinations recognized by the population minimum number of epitope hits / HLA combinations recognized by 90% of the population.

**population/area**	**Class I**			**Class II**			**Class combined**		
	**coverage**	**average_hit**	**pc90**	**coverage**	**average_hit**	**pc90**	**coverage**	**average_hit**	**pc90**
**Central Africa**	59.32%	0.8	0.25	99.1%	3.5	2.2	99.63%	4.31	2.77
**Central America**	2.77%	0.03	0.1	99.98%	2.99	2.16	99.98%	3.02	2.17

**East Africa**	63.48%	0.89	0.27	99.64%	3.78	2.4	99.87%	4.67	3.1
**East Asia**	73.19%	1.05	0.37	95.42%	2.52	1.29	98.77%	3.57	2.09
**Europe**	61.61%	0.85	0.26	99.96%	4.28	3.21	99.98%	5.13	3.74
**North Africa**	54.65%	0.72	0.22	95.74%	2.15	1.23	98.07%	2.87	1.62
**North America**	64.81%	0.92	0.28	100.0%	4.68	3.65	100.0%	5.6	4.25
**Northeast Asia**	54.05%	0.8	0.22	99.51%	3.83	2.43	99.77%	4.63	3.04
**Oceania**	78.85%	1.21	0.47	99.56%	4.07	2.55	99.91%	5.28	3.5
**South Africa**	63.58%	0.89	0.27	5.91%	0.06	0.11	65.74%	0.95	0.29
**South America**	52.65%	0.67	0.21	99.96%	4.49	3.16	99.98%	5.17	3.59
**South Asia**	52.66%	0.7	0.21	99.75%	3.92	2.82	99.88%	4.62	3.22
**Southeast Asia**	66.18%	1.01	0.3	93.99%	2.03	1.15	97.97%	3.04	1.71
**Southwest Asia**	47.77%	0.6	0.19	91.37%	1.81	1.04	95.49%	2.4	1.27
**West Africa**	60.48%	0.82	0.25	99.76%	3.62	2.36	99.91%	4.44	3.01
**West Indies**	53.06%	0.67	0.21	97.3%	2.13	1.31	98.73%	2.8	1.73
**World**	61.01%	0.85	0.26	99.35%	3.97	2.51	99.75%	4.82	3.15
**Average**	**57.07**	**0.79**	**0.26**	**92.72**	**3.17**	**2.09**	**97.26**	**3.96**	**2.6**
**Standard deviation**	**15.56**	**0.24**	**0.08**	**21.84**	**1.18**	**0.91**	**7.96**	**1.23**	**1.01**

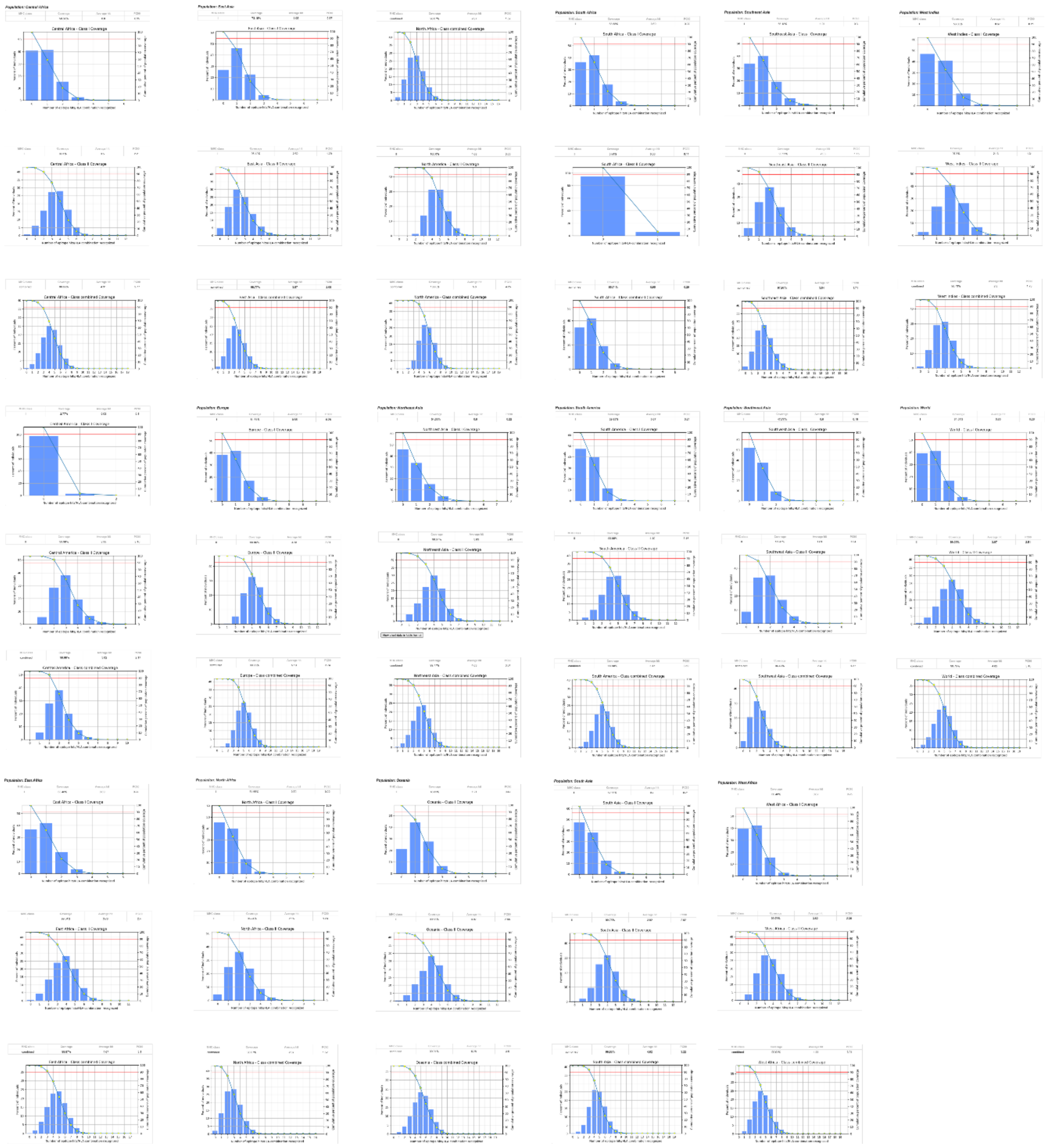

Figure S2. Analysis of population coverage results.

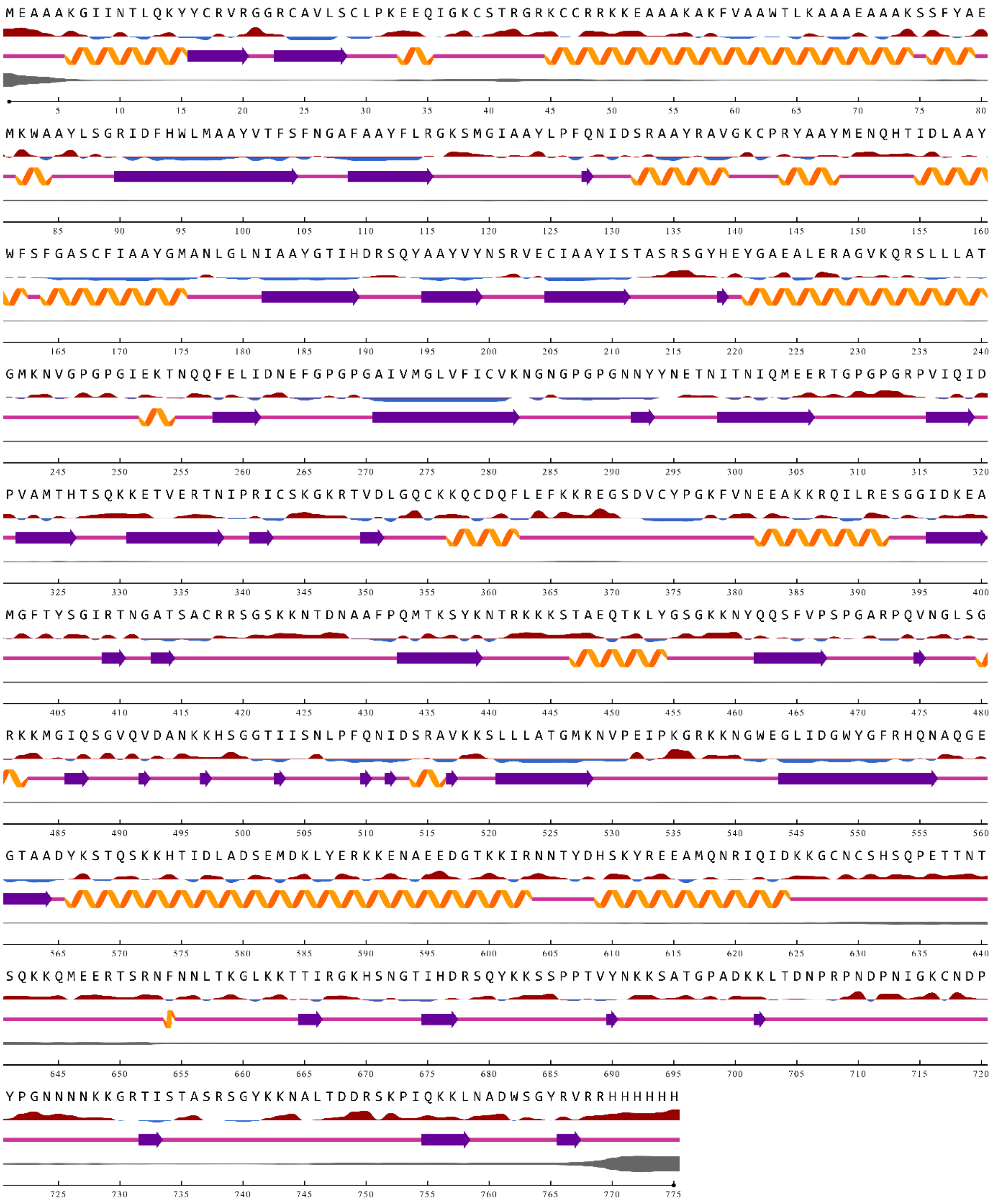

Relative Surface Accessibility: Red is exposed and blue is buried, thresholded at 25%.
Secondary Structure: Helix, Strand, Coil.
Disorder: Thickness of line equals probability of disordered residue.

Figure S3. Predicted H7N9 secondary structure. α-Helices, β-Sheets and Coils are shown in orange, purple and pink colors, respectively.

Table S5. DNA optimization of H7N9-Vac by Codon Adaptation Tool (JCAT) tool

Optimized DNA of H7N9-Vac:
ATGGAAGCAGCTGCGAAAGGAATAATTAATACGCTGCAGAAATACTACTGCCGTGTTCGTGGTGGTCGTTGTGCAG
TGCTGTCTTGTCTGCCGAAAGAGGAACAGATTGGTAAGTGCTCTACTCGCGGTCGTAAGTGCTGCCGTCGCAAAAA
GGAGGCGGCAGCTAAGGCGAAGTTCGTTGCAGCTTGGACCCTGAAAGCGGCGGCTGAGGCGGCAGCGAAATCCTCC
TTTTATGCTGAGATGAAGTGGGCAGCCTACCTGAGCGGTCGTATCGACTTCCATTGGCTGATGGCGGCGTACGTTA
CCTTTAGCTTCAACGGCGCCTTTGCAGCGTACTTCCTGAGAGGCAAGTCTATGGGTATCGCGGCCTACCTCCCGTT
CCAAAACATCGACTCCCGTGCTGCGTACCGCGCGGTGGGCAAGTGCCCGCGTTATGCGGCGTATATGGAAAATCAG
CATACCATCGACCTGGCGGCGTACTGGTTTTCCTTCGGCGCGAGCTGCTTTATTGCGGCCTACGGTATGGCAAACC
TGGGTTTAAATATTGCGGCCTACGGTACGATTCACGACCGTAGTCAATATGCAGCGTACGTGTATAACAGCCGTGT
CGAGTGCATTGCTGCTTACATTAGCACCGCTAGCCGCTCTGGCTATCACGAGTATGGTGCGGAAGCGTTAGAACGG
GCCGGTGTGAAACAACGCTCCCTGTTGTTGGCTACTGGTATGAAAAATGTTGGTCCGGGTCCGGGCATTGAAAAAA
CCAATCAGCAGTTTGAACTCATCGACAACGAATTTGGTCCGGGTCCTGGCGCGATTGTGATGGGTTTGGTTTTTAT
CTGTGTTAAAAACGGAAATGGTCCGGGTCCAGGTAACAACTATTACAACGAGACTAACATAACCAACATCCAAATG
GAAGAGCGTACGGGGCCAGGTCCGGGTCGCCCAGTCATTCAAATTGACCCGGTGGCGATGACCCACACCTCGCAAA
AAAAAGAAACCGTTGAACGAACGAACATCCCGCGTATTTGCAGCAAAGGCAAACGTACCGTCGATTTGGGTCAATG
TAAAAAGCAGTGTGATCAGTTCCTGGAGTTCAAAAAGCGCGAGGGTAGCGATGTTTGTTATCCGGGTAAATTCGTG
AATGAGGAGGCGAAGAAGAGGCAGATCCTGCGTGAGAGCGGTGGTATTGACAAGGAGGCTATGGGCTTCACCTATA
GCGGTATCCGCACGAACGGCGCGACCAGCGCGTGCCGTCGTTCCGGCAGCAAAAAGAACACCGACAACGCGGCTTT
CCCGCAAATGACCAAATCCTATAAAAACACCCGTAAAAAGAAGTCTACGGCAGAGCAAACCAAATTGTACGGCTCT
GGCAAGAAGAACTACCAGCAAAGCTTTGTCCCGTCTCCGGGTGCTCGTCCACAGGTAAATGGTCTGAGCGGACGGA
AGAAGATGGGTATCCAATCTGGCGTGCAGGTTGACGCAAATAAAAAGCACAGCGGTGGCACCATTATTTCTAATTT
GCCGTTCCAGAACATCGACAGCAGAGCCGTCAAAAAGTCCCTGTTGCTGGCGACCGGTATGAAGAACGTGCCGGAA
ATTCCGAAAGGCCGCAAGAAAAACGGCTGGGAAGGTCTGATCGACGGTTGGTATGGCTTTCGTCATCAGAACGCGC
AGGGTGAAGGCACAGCTGCGGACTACAAATCGACCCAGAGCAAGAAACACACCATAGACCTCGCAGATAGCGAAAT
GGATAAACTGTACGAACGCAAGAAGGAGAACGCTGAAGAGGATGGTACGAAAAAGATCCGTAACAATACCTACGAC
CACTCCAAGTATCGTGAGGAGGCGATGCAGAATCGTATCCAAATCGATAAAAAAGGCTGCAACTGCTCCCATTCAC
AGCCGGAGACGACCAACACCTCCCAGAAGAAGCAAATGGAAGAACGCACCAGTCGTAATTTTAATAACCTTACCAA
AGGTCTGAAAAAGACCACGATCAGAGGTAAACACTCAAATGGTACGATCCACGATCGCAGCCAATATAAGAAGTCT
AGCCCGCCTACAGTTTACAACAAGAAGTCCGCGACCGGTCCGGCAGATAAAAAACTGACCGATAACCCGCGTCCGA
ATGATCCGAATATCGGCAAGTGCAATGATCCGTATCCGGGCAACAACAACAATAAAAAGGGTCGTACCATCAGTAC
TGCGAGCCGTAGCGGTTATAAGAAGAACGCCCTGACCGACGACCGTAGCAAACCGATTCAGAAGAAACTGAATGCG
GATTGGTCGGGTTACCGCGTGCGACGTCTCGAGCACCACCACCACCACCACTGA

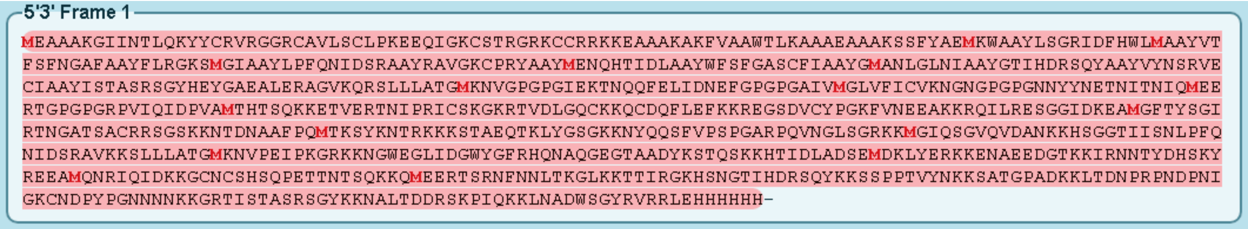

Figure S4. Construct Open reading frames in https://web.expasy.org/translate/. Open reading frames are highlighted in red.

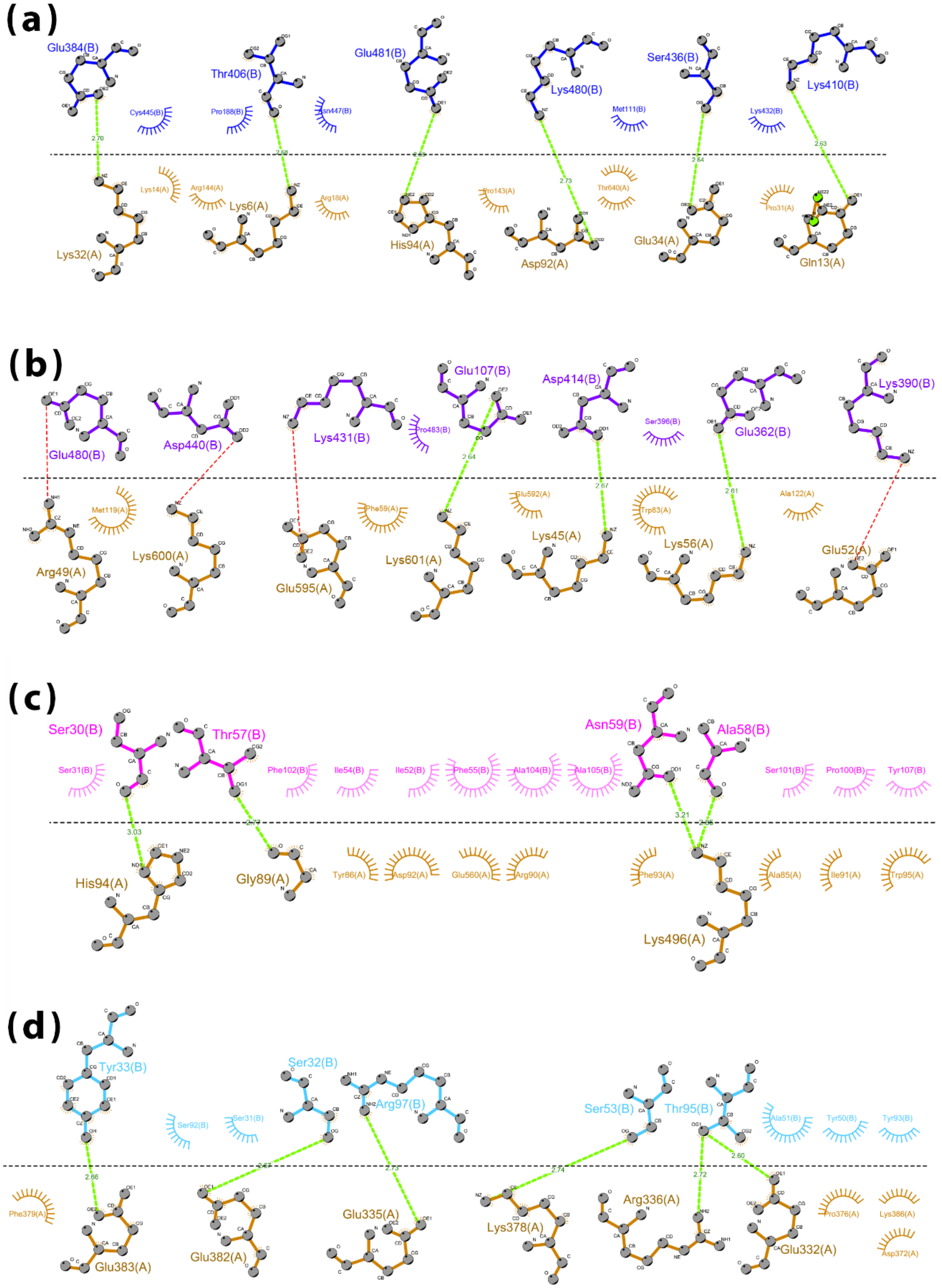

Figure S5. (a), (b), (c) and (d) 2D interaction of residues in H7N9v-TLR7, H7N9v-TLR8, H7N9vac-m826 Heavy chain and H7N9vac-m826 Light chain complex, respectively.

The results and graphics presented below are based on water refined models generated by HADDOCK. The clusters (indicated in color in the graphs) are calculated based on the interface-ligand RMSDs calculated by HADDOCK, with the interface defined automatically based on all observed contacts. The various structural analysis (FCC, i-RMSD and l-RMSD) are made with respect to the best HADDOCK model (the one with the lowest HADDOCK score).

i-RMSD -> interface-RMSD calculated on the backbone (CA, C, N, O, P) atoms of all residues involved in intermolecular contact using a 10Å cutoff.

l-RMSD -> ligand-RMSD calculated on the backbone atoms (CA, C, N, O, P) of all (N>1) molecules after fitting on the backbone atoms of the first (N=1) molecule.

FCC -> Fraction of common contacts. The intermolecular contacts are defined based on the best HADDOCK model using a 5Å cutoff.

a.u. -> Arbitrary Units

The cluster averages and standard deviations are indicated by colored dots with associated error bars. The average values are calculated on the best 4 structures of each cluster (based on the HADDOCK score).

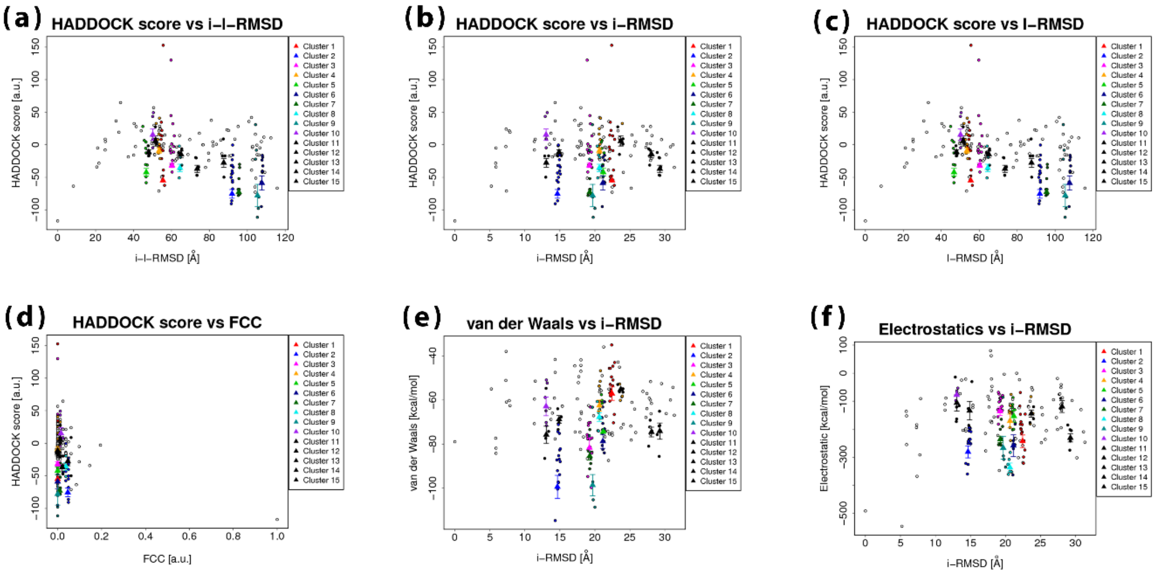

Figure S6. Results analysis related to HADDOCKH run of the H7N9v-TLR7 complex. (a) HADDOCK score vs i-l-RMSD. (b) HADDOCK score vs i-RMSD. (c) HADDOCK score vs l-RMSD. (d) HADDOCK score vs FFC. (e) van der Waals vs i-RMSD. (f) Electrostatics vs i-RMSD.

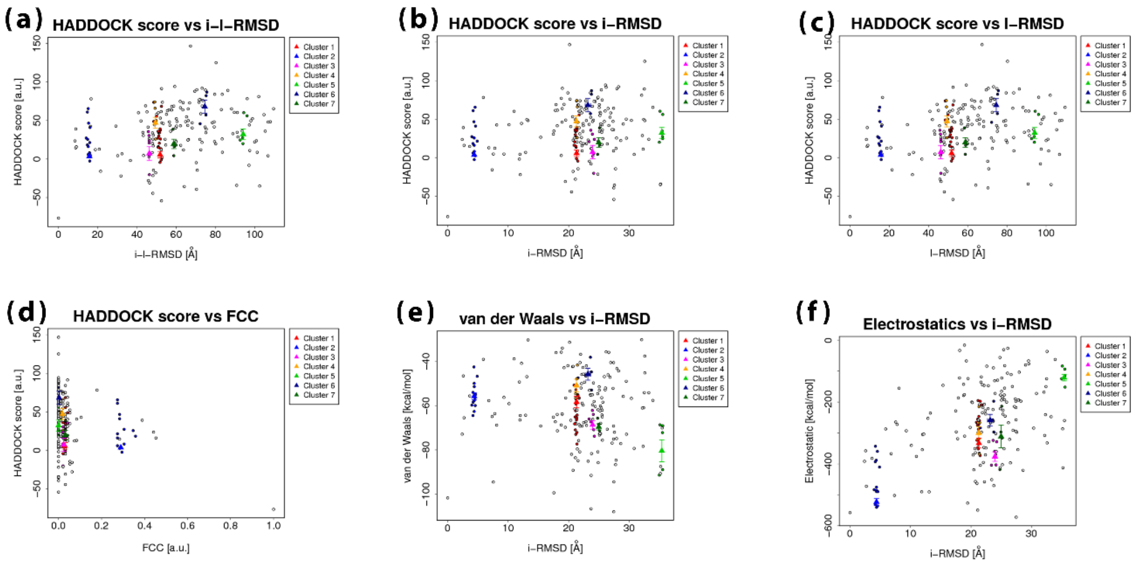

Figure S7. Results analysis related to HADDOCKH run of the H7N9v-TLR8 complex. (a) HADDOCK score vs i-l-RMSD. (b) HADDOCK score vs i-RMSD. (c) HADDOCK score vs l-RMSD. (d) HADDOCK score vs FFC. (e) van der Waals vs i-RMSD. (f) Electrostatics vs i-RMSD.

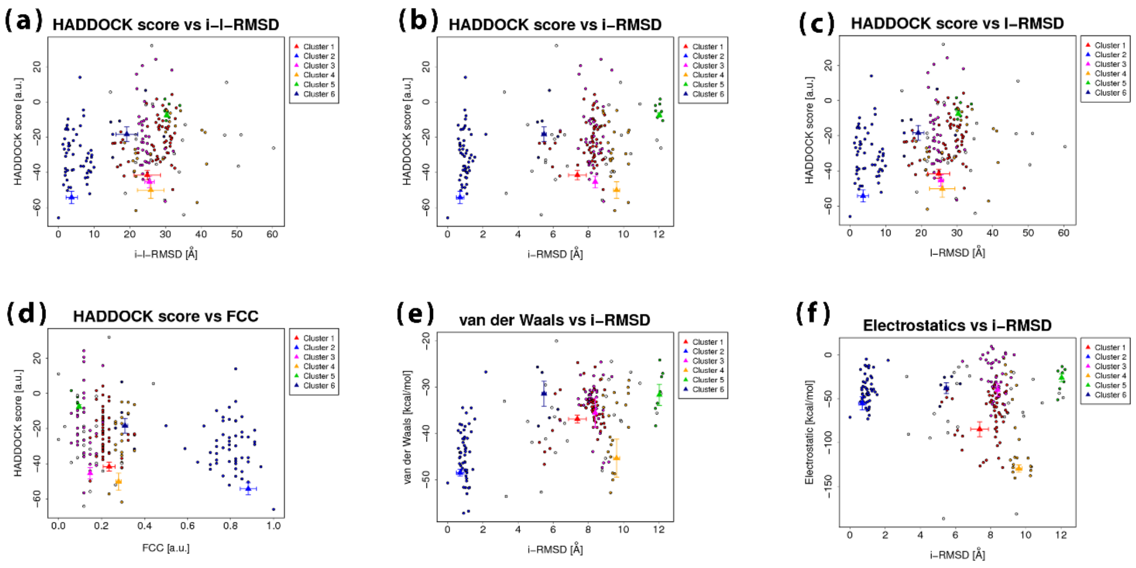

Figure S8. Results analysis related to HADDOCKH run of the H7N9v- m826 Heavy chain complex. (a) HADDOCK score vs i-l-RMSD. (b) HADDOCK score vs i-RMSD. (c) HADDOCK score vs l-RMSD. (d) HADDOCK score vs FFC. (e) van der Waals vs i-RMSD. (f) Electrostatics vs i-RMSD.

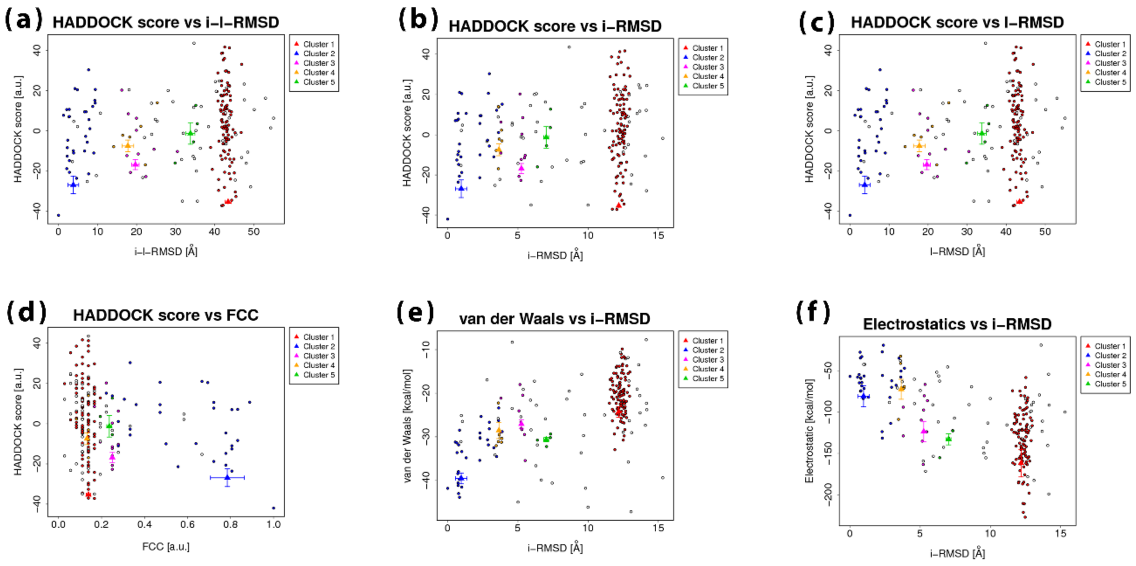

Figure S9. Results analysis related to HADDOCKH run of the H7N9v-m826 Light chain complex. (a) HADDOCK score vs i-l-RMSD. (b) HADDOCK score vs i-RMSD. (c) HADDOCK score vs l-RMSD. (d) HADDOCK score vs FFC. (e) van der Waals vs i-RMSD. (f) Electrostatics vs i-RMSD.

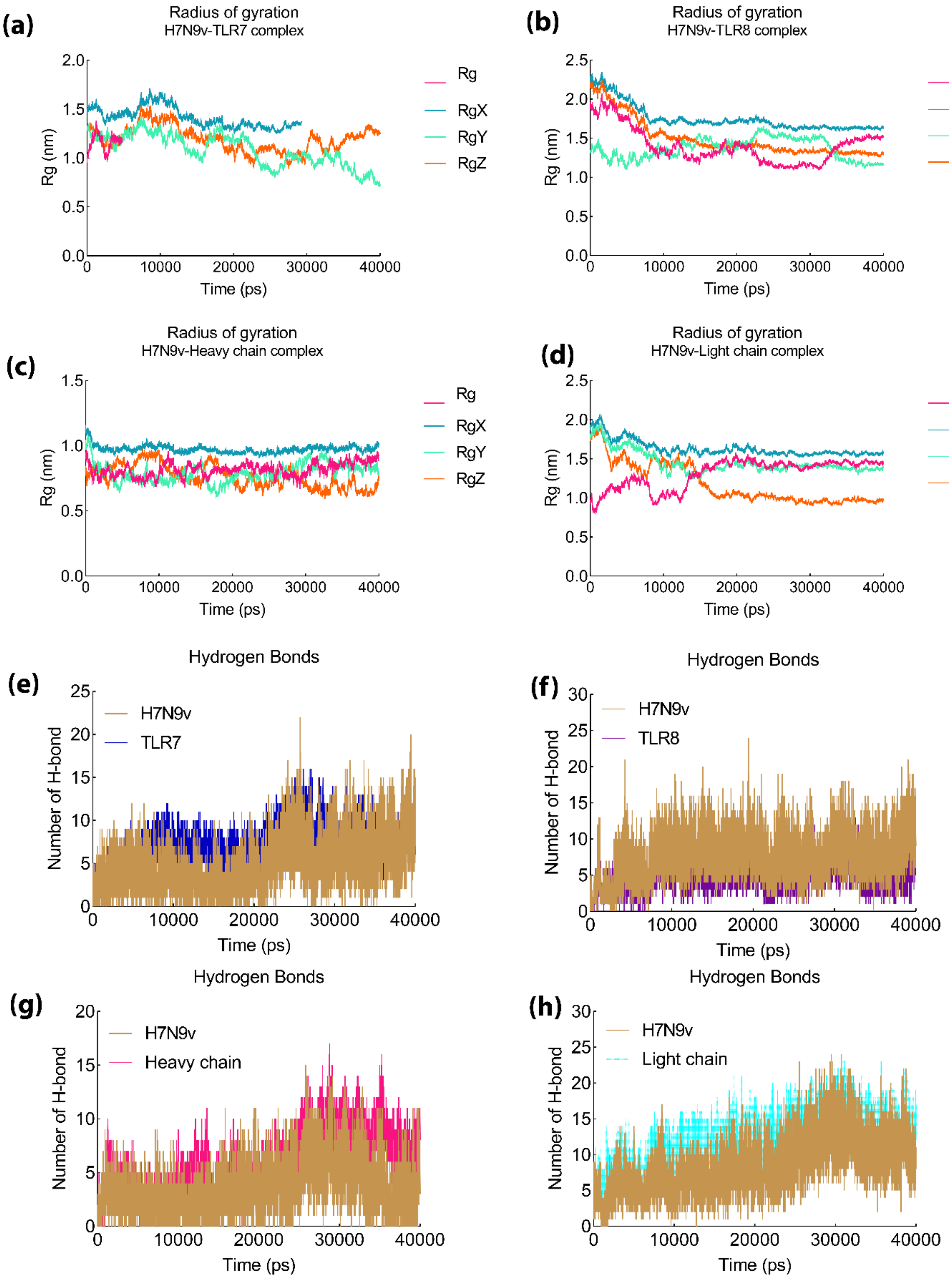

Figure S10. MD simulation Gyrations and Hydrogen bonds results of H7N9v with proteins complex. (a) Radius of Gyration graph of H7N9vac-TLR7, (b) Radius of Gyration graph

H7N9vac-TLR8 complexes. (c) Radius of Gyration graph of H7N9vac-m826 Heavy chain, and (d) Radius of Gyration graph of H7N9vac-m826 Light chain complexes. (e) (f), (j) and (h) H-Bond graph of H7N9vac-TLR7, H7N9vac-TLR8, H7N9vac-m826 Heavy chain and H7N9vac-m826 Light chain complexes.
